# The lactate-NAD^+^ axis activates cancer-associated fibroblasts by downregulating p62

**DOI:** 10.1016/j.celrep.2022.110792

**Published:** 2022-05-10

**Authors:** Juan F. Linares, Tania Cid-Diaz, Angeles Duran, Marta Osrodek, Anxo Martinez-Ordoñez, Miguel Reina-Campos, Hui-Hsuan Kuo, Olivier Elemento, M. Laura Martin, Thekla Cordes, Timothy C. Thompson, Christian M. Metallo, Jorge Moscat, Maria T. Diaz-Meco

**Affiliations:** 1 Department of Pathology and Laboratory Medicine, Weill Cornell Medicine, New York, NY 10065, USA; 2 Sanford Burnham Prebys Medical Discovery Institute, La Jolla, CA 92037, USA; 3 Englander Institute for Precision Medicine, Weill Cornell Medicine, New York, NY 10065, USA; 4 Department of Bioengineering, University of California, San Diego, La Jolla, CA 92093, USA; 5 Department of Genitourinary Medical Oncology, The University of Texas MD Anderson Cancer Center, Houston, TX, USA; 6 Molecular and Cell Biology Laboratory, Salk Institute for Biological Studies, La Jolla, CA 92037, USA; 7 These authors contributed equally; 8 Lead contact

## Abstract

Reduced p62 levels are associated with the induction of the cancer-associated fibroblast (CAF) phenotype, which promotes tumorigenesis *in vitro* and *in vivo* through inflammation and metabolic reprogramming. However, how p62 is downregulated in the stroma fibroblasts by tumor cells to drive CAF activation is an unresolved central issue in the field. Here we show that tumor-secreted lactate downregulates p62 transcriptionally through a mechanism involving reduction of the NAD^+^/NADH ratio, which impairs poly(ADP-ribose)-polymerase 1 (PARP-1) activity. PARP-1 inhibition blocks the poly(ADP-ribosyl)ation of the AP-1 transcription factors, c-FOS and c-JUN, which is an obligate step for p62 downregulation. Importantly, restoring p62 levels in CAFs by NAD^+^ renders CAFs less active. PARP inhibitors, such as olaparib, mimick lactate in the reduction of stromal p62 levels, as well as the subsequent stromal activation both *in vitro* and *in vivo*, which suggests that therapies using olaparib would benefit from strategies aimed at inhibiting CAF activity.

## In brief

Linares et al. describe a molecular mechanism for cancer-associated fibroblast activation. Tumor-secreted lactate downregulates p62 in the stroma by reducing NAD^+^, which impairs PARP-1 activity, blocking AP-1-mediated p62 transcription. PARP-1 inhibitors mimic lactate to induce stroma activation, suggesting that anti-stromal therapies might enhance their efficacy.

## Graphical Abstract

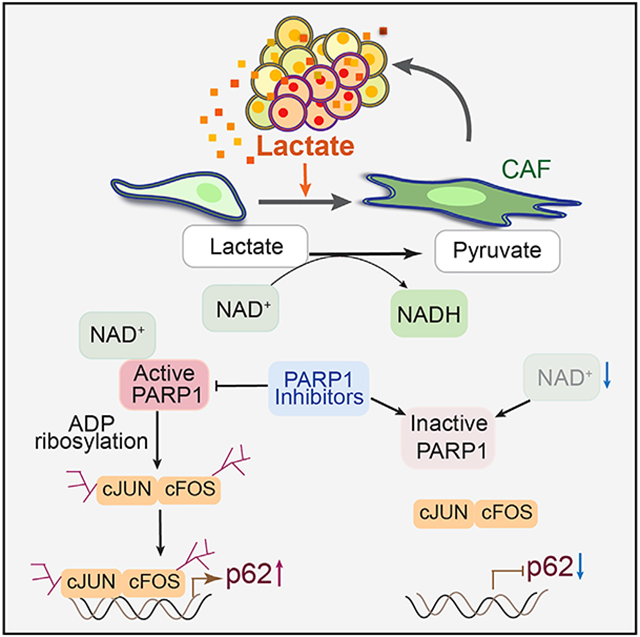

## INTRODUCTION

Mounting evidence identifies the tumor microenvironment (TME), including stromal fibroblasts, as a driver of tumorigenesis in cancer (Klemm and Joyce, 2015; Nakanishi et al., 2018; Zhang et al., 2013, 2020). It is well accepted now that the activation of tumor stromal fibroblasts to a state commonly known as cancer-associated fibroblasts (CAFs) can embolden epithelial cancer cells to progress to more malignant stages and affect disease outcomes (Barron and Rowley, 2012; Cunha et al., 2002). CAFs impact the tumor epithelium by modulating many functions through the secretion of stromal growth factors and inflammatory mediators, by reprogramming their metabolism to provide nutrients and survival factors, and by remodeling the extracellular matrix (ECM) (Hanahan and Weinberg, 2011; Kasashima et al., 2020; Sahai et al., 2020). This fibroblast-epithelium synergistic crosstalk promotes malignancy and is believed to negatively impact cancer treatment by promoting the accumulation of “persisting” or “resistant” tumor cells, which limit therapy efficacy and promote resistance (Bluemn et al., 2017; Linares et al., 2017; Zhang et al., 2020). The renewed interest in the study of CAFs has resulted in the extensive characterization of their cell heterogeneity, as well as the establishment of a complex catalog of the myriad of secreted intermediates that likely impact tumor progression and response to therapy. However, a major gap in the field is the identification of the key master regulators of the acquisition of the CAF phenotype, as well as the molecular mechanisms whereby the tumor epithelium drives this process.

Our laboratory has demonstrated that the autophagy substrate and signaling adaptor p62 (encoded by *SQSTM1* gene) is a critical regulator of CAF biology (Huang et al., 2018; Linares et al., 2017; Valencia et al., 2014). p62 has a dual role in cancer (Duran et al., 2008, 2011, 2016; Moscat et al., 2016; Umemura et al., 2016). Although it plays an oncogenic function in the tumor epithelium, it is a suppressor of tumor progression by restraining CAF activation (Duran et al., 2016; Goruppi and Dotto, 2013; Linares et al., 2017; Reina-Campos et al., 2018; Valencia et al., 2014). p62 is upregulated in the epithelium of many types of tumors, which invariably associates with more aggressive cancer and poorer overall survival (Moscat and Diaz-Meco, 2009; Moscat et al., 2016). However, many tumors display reduced levels of p62 in their stroma, especially in CAFs (Duran et al., 2016; Goruppi and Dotto, 2013; Linares et al., 2017; Valencia et al., 2014). Our laboratory has demonstrated that p62 downregulation is a central event in the acquisition of the CAF phenotype and in creating a pro-tumorigenic microenvironment conducive to cancer (Linares et al., 2017; Valencia et al., 2014). p62 inactivation in prostate stromal fibroblasts impairs their metabolic detoxification capacity and leads to the accumulation of reactive oxygen species, which in turn results in the release of pro-survival inflammatory cytokines (Valencia et al., 2014). Under nutrient stress conditions, p62 downregulation in fibroblasts promotes tumorigenesis by enabling their own survival and that of tumor epithelial cells through the secretion of asparagine (Linares et al., 2017). These studies highlight the pivotal role of p62 as a tumor suppressor in the stroma by activating pro-survival pathways and the cellular adaptation to nutrient deprivation.

Therefore, whereas the genetic inactivation of p62 selectively in the tissue epithelium impairs tumorigenesis, that in the stromal fibroblasts has the opposite effect by impacting several metabolic and inflammatory pathways (Moscat et al., 2016). The fact that whole-body genetic inactivation of p62 results in increased tumorigenesis, mimicking the phenotype of mice with p62 deficiency in the fibroblast stromal compartment, strongly suggests that the stromal role of p62 as a tumor suppressor is what determines the global impact of p62 in cancer (Valencia et al., 2014). This indicates that the tumor-promoting signals produced by the loss of p62 in CAFs override the requirement for p62 in the tumor epithelium, which highlights the importance of studying how p62 is lost in the tumor’s fibroblast compartment. In this regard, a key pending question is to determine how tumors downregulate p62 in the stroma to promote the CAF phenotype. Identifying this mechanism is of paramount importance because, given the critical role of p62 in CAF activation and function, this will provide a paradigm for our understanding of the master regulators of stromal activation, which will be more amenable to therapeutic intervention than trying to affect the complex downstream effectors unleashed upon stromal activation.

Here we investigated the mechanisms whereby cancer cells promote the acquisition of the pro-tumorigenic CAF phenotype. We show that the lactate secreted by tumor cells downregulates p62 at the transcriptional level to induce stromal activation. Lactate caused a reduction in the NAD^+^/NADH ratio that impaired poly(ADP-ribose)polymerase 1 (PARP-1) activity. The fact that PARP-1 inhibitors used in the clinic, such as olaparib, mimicked the effect of lactate in downregulating p62 and promoting CAF activity reveals an unanticipated potential weakness of therapies based on PARP-1 inhibitors and suggests that combination treatment of olaparib with anti-stromal targeted therapies would improve olaparib efficacy.

## RESULTS

### Prostate cancer cells secrete a soluble factor that reduces p62 expression in stromal fibroblasts

**Figure 1.**
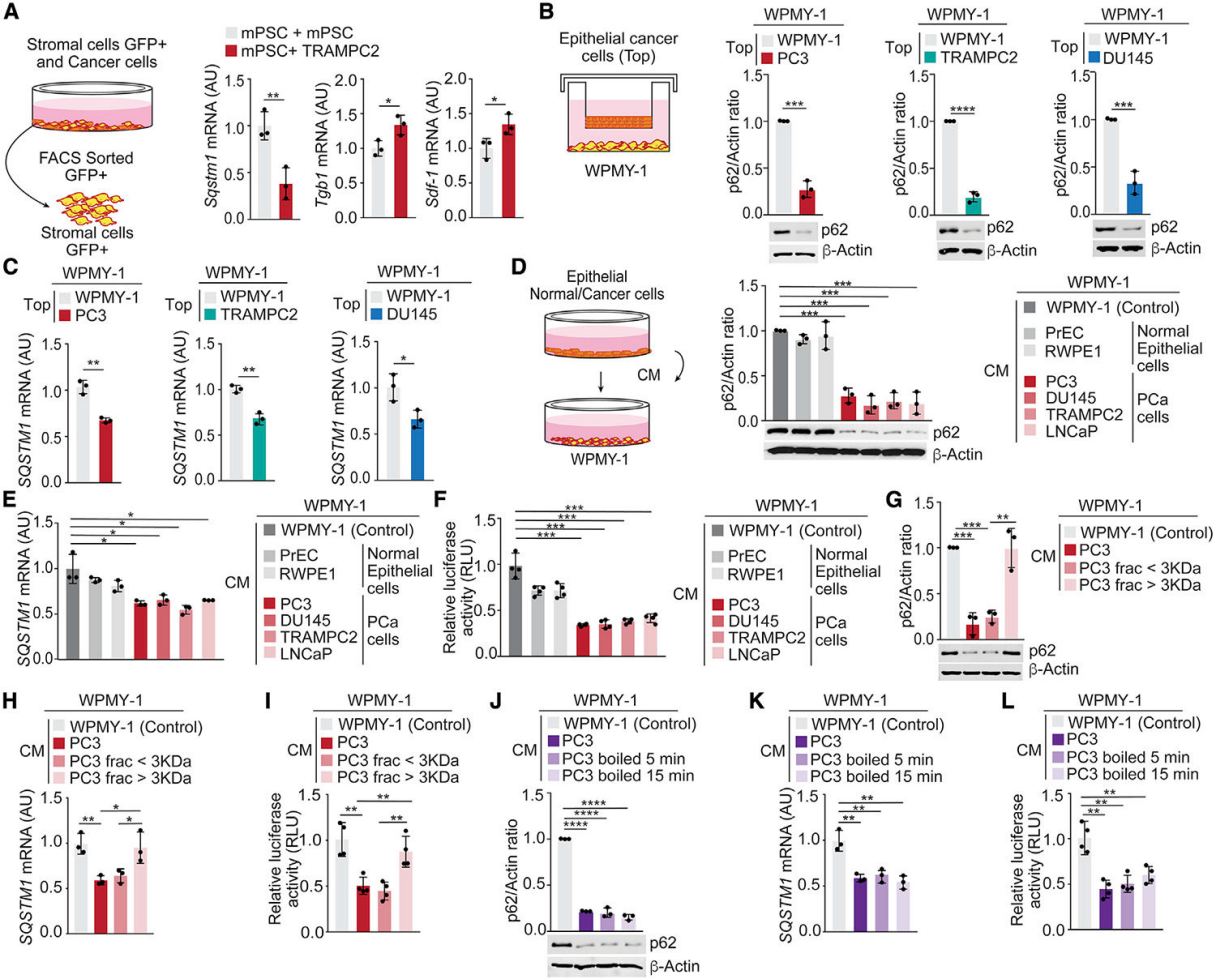
PCa cells secrete a soluble factor that reduces p62 expression in stromal cells (A) Experimental design and qPCR of the indicated genes in mPSC-GFP cells mixed with TRAMPC2 cells and cultured for 7 days (n = 3 biological replicates). (B and C) Experimental design, immunoblot analysis (B), and qPCR of *SQSTM1* (C) in WPMY-1 cells co-cultured with PCa cells during 48 h (n = 3 biological replicates). (D and E) Experimental design, immunoblot analysis (D), and qPCR of *SQSTM1* in WPMY-1 cells incubated 48 h with conditioned media (CM) from PCa cells (E) (n = 3 biological replicates). (F) *SQSTM1* promoter-driven luciferase in WPMY-1 cells incubated 48 h with CM from PCa cells (n = 3 biological replicates). (G and H) Immunoblot analysis (G) and qPCR of *SQSTM1* (H) in WPMY-1 cells incubated 48 h with fractionated CM from PC3 cells (n = 3 biological replicates). (I) *SQSTM1* promoter-driven luciferase in WPMY-1 cells treated as in (G) (n = 3 biological replicates). (J and K) Immunoblot analysis (J) and qPCR of *SQSTM1* (K) in WPMY-1 cells incubated 48 h with normal or boiled CM from PC3 cells (n = 3 biological replicates). (L) *SQSTM1* promoter-driven luciferase in WPMY-1 cells treated as in (J) (n = 3 biological replicates). Results are shown as mean ± SEM. *p < 0.05, **p < 0.01, ***p < 0.001, ****p < 0.0001.

To investigate how p62 expression is downregulated in the tumor stroma, we set up an *in vitro* cell system of mouse GFP-labeled prostate stromal cells (mPSCs) mixed with TRAMPC2 prostate cancer (PCa) epithelial cells. Analysis of GFP-positive stromal cells isolated by fluorescence-activated cell sorting (FACS) showed reduced *Sqstm1* (coding for p62) mRNA levels in stromal cells on incubation with tumor cells, which also correlated with an increase in bona fide CAF markers, such as *Tgfb1* and *Sdf-1* (Figure 1A). To test whether the downregulation of p62 required cell contact between tumor and stromal cells, we co-cultured human prostate stromal fibroblasts (WPMY-1) with different PCa cell lines in a double-chamber setting. p62 was also downregulated at the protein and mRNA levels in stromal cells under these conditions (Figures 1B and 1C), indicating that a soluble factor secreted by PCa epithelial cells was sufficient to downregulate p62 in stromal cells. In fact, incubation of stromal cells with conditioned media (CM) from PCa cells effectively reduced p62 protein and mRNA levels in stromal cells, without any effect by the CM from normal prostate epithelial cells (PrEC or RWEP1) (Figures 1D and 1E). To determine whether the reduction in *SQSTM1* mRNA was due to inhibited transcriptional activity, we used a luciferase reporter under the control of *SQSTM1* promoter (Duran et al., 2008). PCa CM reduced the activity of the luciferase reporter (Figure 1F), suggesting that stromal p62 downregulation is mediated through the repression of *SQSTM1* promoter by a soluble factor secreted by tumor cells.

To identify this putative tumor-derived soluble factor, CM from PC3 cells were size fractionated. Both the unfractionated tumor CM and the <3-kDa fraction comparably downregulated p62 at the protein and mRNA levels in fibroblasts (Figures 1G and 1H), as well as the activity of the *SQSTM1* promoter-driven luciferase reporter (Figure 1I). However, the >3-kDa fraction was totally inactive in these assays (Figures 1G-1I). The active soluble factor was heat stable (Figures 1J-1L), which suggested that it could be a metabolite.

### Lactate downregulates p62 in stromal fibroblasts

**Figure 2.**
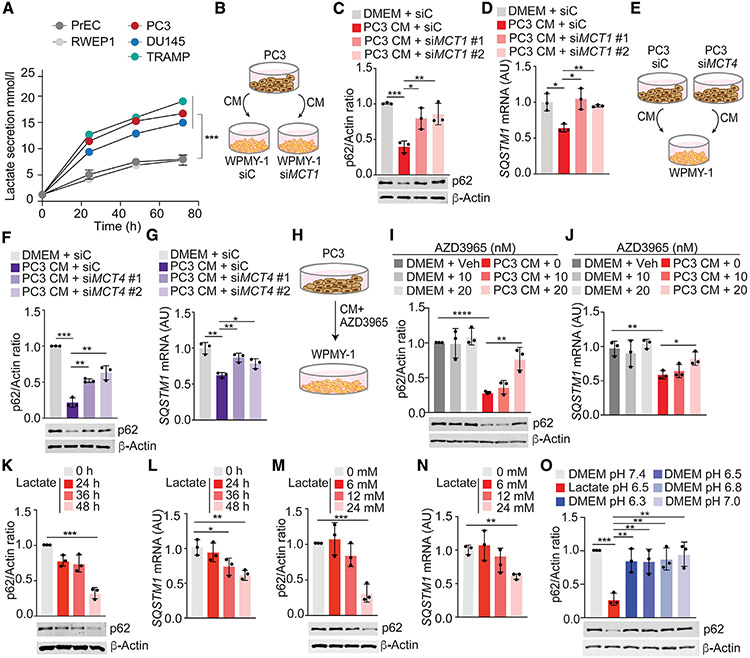
Lactate is sufficient to downregulate p62 (A) Lactate secretion was determined by YSI2950 analyzer from PCa cells for 72 h. Results are normalized to total protein content (n = 3 biological replicates). (B–D) Experimental design (B), immunoblot analysis (C), and qPCR of *SQSTM1* (D) in WPMY-1 cells, transduced with the indicated siRNAs and incubated with CM from PC3 cells for 48 h (n = 3 biological replicates). (E–G) Experimental design (E), immunoblot analysis (F), and qPCR of *SQSTM1* (G) in WPMY-1 cells incubated with CM from PC3 cells, transduced with the indicated siRNAs, for 48 h (n = 3 biological replicates). (H–J) Experimental design (H), immunoblot analysis (I), and qPCR of *SQSTM1* (J) in WPMY-1 cells, treated with AZD3965 and incubated with CM from PC3 cells for 48 h (n = 3 biological replicates). (K and L) Immunoblot analysis (K) and qPCR of *SQSTM1* (L) in WPMY-1 cells incubated with lactate (24 mM) at different times (n = 3 biological replicates). (M and N) Immunoblot analysis (M) and qPCR of *SQSTM1* (N) in WPMY-1 cells incubated with different doses of lactate for 48 h (n = 3 biological replicates). (O) Immunoblot analysis in WPMY-1 cells incubated with lactate 24 mM (pH 6.5) or DMEM at different pH values for 48 h (n = 3 biological replicates). Results are shown as mean ± SEM. *p < 0.05, **p < 0.01, ***p < 0.001, ****p < 0.0001. See also Figure S1.

Because cancer cells secrete large amounts of lactate that accumulates in the TME as a consequence of the Warburg effect (Vander Heiden et al., 2009), we speculated that lactate could be the metabolite responsible for p62 downregulation. As expected, PCa cell lines secreted a higher amount of lactate than normal prostate epithelial cells (Figure 2A). Because the cellular uptake of lactate is mediated by different monocarboxylate transporters (MCT1, MCT2, MCT3, and MCT4) (Halestrap, 2013), we next tested whether blocking these transporters could impair lactate-induced p62 downregulation. Knockdown of *MCT1*, which controls lactate cellular import in WPMY-1 cells, resulted in the inhibition of p62 downregulation induced by CM from PC3 cells (Figures 2B-2D and S1A). Likewise, p62 downregulation in WPMY-1 cells by the same CM was abrogated when *MCT4*, which controls lactate export, was knocked down in PC3 cells (Figures 2E-2G and S1B). Treatment with the MCT1 inhibitor, AZD3965, impaired p62 downregulation in WPMY-1 cells treated with PC3 CM (Figures 2H-2J). These results demonstrate that secretion of lactate by PCa cells and its uptake by stromal cells is a critical step in stromal p62 downregulation.

To demonstrate that lactate is sufficient to downregulate p62 in fibroblasts, we incubated WPMY-1 cells with different concentrations of lactate for 24 or 48 h, which resulted in p62 downregulation in a time- and dose-dependent manner (Figures 2K-2N). The amount of lactate necessary to downregulate p62 in stromal cells (Figures 2M and 2N) was similar to that secreted by PCa cells (Figure 2A) and to that reported from tumors *in vivo* (Walenta et al., 2000). MCTs are proton-coupled symporters, which explains why lactate-mediated p62 downregulation was dependent on an acidic pH (Figure S1C). However, the simple acidification of the culture media was not sufficient to downregulate p62 (Figure 2O). Lactate-mediated p62 downregulation was observed in multiple primary fibroblasts from different origins (Figures S1D-S1G). No changes in p62 levels were observed upon lactate treatment of PCa epithelial cells (Figure S1H). To rule out the potential contribution of a hypothetical role of p62 protein stability in the response to lactate, we incubated cycloheximide-treated cells with lactate and analyzed p62 protein levels. Lactate did not affect p62 stability (Figure S1I). Inhibition of the proteasome, autophagy, or cysteine proteases did not rescue lactate-mediated p62 downregulation (Figures S1J-S1N). These results demonstrate that lactate secreted by epithelial PCa cells impaired p62 expression in stromal cells at the transcriptional level.

### AP-1 controls p62 downregulation by lactate

**Figure 3.**
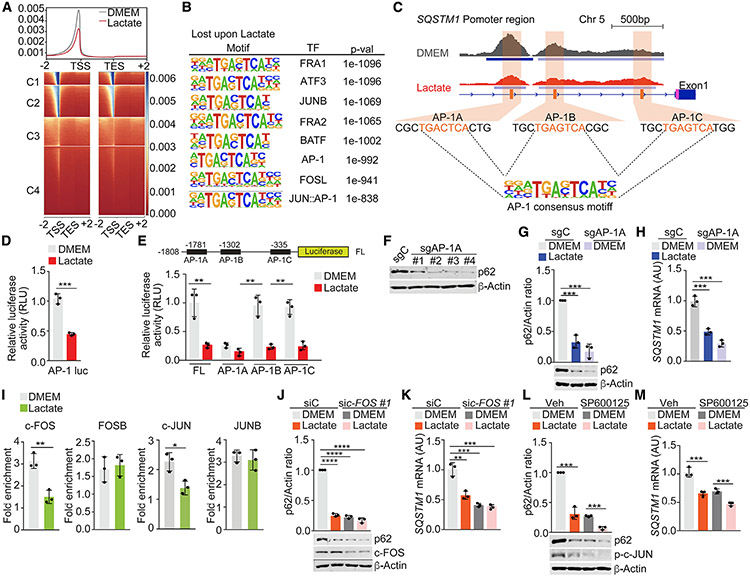
AP-1 elements control p62 downregulation by lactate (A) Four-cluster (C1, C2, C3, and C4) heatmap of ATAC-seq peaks, ±2 kb from transcription start site (TSS) in WPMY-1 cells treated or not with lactate for 48 h. (B) HOMER discovery analysis of transcription factor (TF)-enriched promoter regions (1–2 kb) closed in WPMY-1 cells treated with lactate (control versus lactate). (C) Genome browserview at *SQSTM1* promoter. Chromatin accessibility (ATAC-seq) and RNA-seq profiles are shown in WPMY-1 cells treated or not with lactate. (D) AP-1-driven luciferase in WPMY-1 cells treated with lactate for 48 h (n = 3 biological replicates). (E) *SQSTM1* promoter-driven luciferase in WPMY-1 cells, transduced with the indicated plasmids, and treated with lactate for 48 h (n = 3 biological replicates). (F) Immunoblot analysis in sgC and sgAP-1A WPMY-1 cells for the indicated proteins. (G and H) Immunoblot analysis (G) and qPCR of *SQSTM1* (H) in sgC and sgAP-1A WPMY-1 cells incubated with lactate (n = 3 biological replicates). (I) ChIP-PCR analysis of *SQSTM1* promoter (AP-1A) occupancy of c-JUN, c-FOS, FOSB, or JUNB in WPMY-1 cells treated or not with lactate (24 mM) for 48 h (n = 3 biological replicates). (J and K) Immunoblot analysis (J) and *SQSTM1* mRNA levels (K) in WPMY-1 cells incubated with lactate and transduced with the indicated siRNAs (n = 3 biological replicates). (L and M) Immunoblot analysis (L) and qPCR of *SQSTM1* in WPMY-1 cells incubated with lactate and treated with 10 μM SP600125 (M) (n = 3 biological replicates). Results are shown as mean ± SEM. *p < 0.05, **p < 0.01, ***p < 0.001, ****p < 0.0001. See also Figure S2.

To unravel the mechanisms whereby lactate downregulates stromal p62, we next investigated the chromatin accessibility landscape of lactate-treated fibroblasts by genome-wide assay for transposase-accessible chromatin with sequencing (ATAC-seq). This analysis revealed a widespread decrease in chromatin accessibility in lactate-treated cells (Figure 3A). HOMER (Hypergeometric Optimization of Motif EnRichment) analysis showed a significant enrichment of AP-1 transcription factors in the closed chromatin regions of lactate-treated cells (Figure 3B). Footprinting analysis predicted the decreased occupancy of AP-1 family factors in lactate-treated cells (Figures S2A and S2B). Analysis of *SQSTM1* promoter demonstrated the presence of three AP-1 regulatory sites that also showed reduced chromatin accessibility under lactate-treated conditions (Figures 3C and S2C). Consistently, lactate repressed an AP-1-driven luciferase reporter (Figure 3D), which collectively indicated that the repression of AP-1 sites in the *SQSTM1* promoter could account for lactate effects on stromal p62 expression.

To establish the contribution of the AP-1 sites in the *SQSTM1* promoter, we individually mutated them (AP-1A, AP-1B, and AP-1C) and determined the impact of these mutations on *SQSTM1* promoter activity. Mutation of AP-1A completely inactivated *SQSTM1* promoter to levels comparable with those produced by lactate, whereas mutation of AP-1B or AP-1C had no effect (Figure 3E), suggesting that AP-1A is the critical enhancer element in the *SQSTM1* promoter to mediate lactate repression. To test this hypothesis, we selectively deleted the AP-1A element in the endogenous *SQSTM1* promoter by CRISPR-Cas9 editing to generate four independent WPMY-1 cell cultures (sgAP-1A), which resulted in the inhibition of p62 mRNA and protein expression (Figures 3F-3H). These results establish the relevance of AP-1A for the regulation of p62 expression in stromal cells. In keeping with this notion, chromatin immunoprecipitation (ChIP) analysis demonstrated that the recruitment of c-FOS and c-JUN, but not of FOSB or JUNB, to the AP-1A regulatory site was significantly decreased by lactate with no changes detected in binding to AP-1B or AP-1C sites (Figures 3I and S2D). Consistently, the downregulation of c-FOS, c-JUN, and JNK, or the inhibition of c-JUN phosphorylation with two pharmacological inhibitors of JNK mimicked the effect of lactate on p62 expression both at the protein and the mRNA levels (Figures 3J-3M and S2E-S2L). These data indicate that the impaired recruitment of a c-JUN/c-FOS AP-1 transcriptional complex plays a critical role in lactate-induced stromal p62 downregulation.

### Reduction in NAD^+^ levels by lactate metabolism mediates p62 downregulation

**Figure 4.**
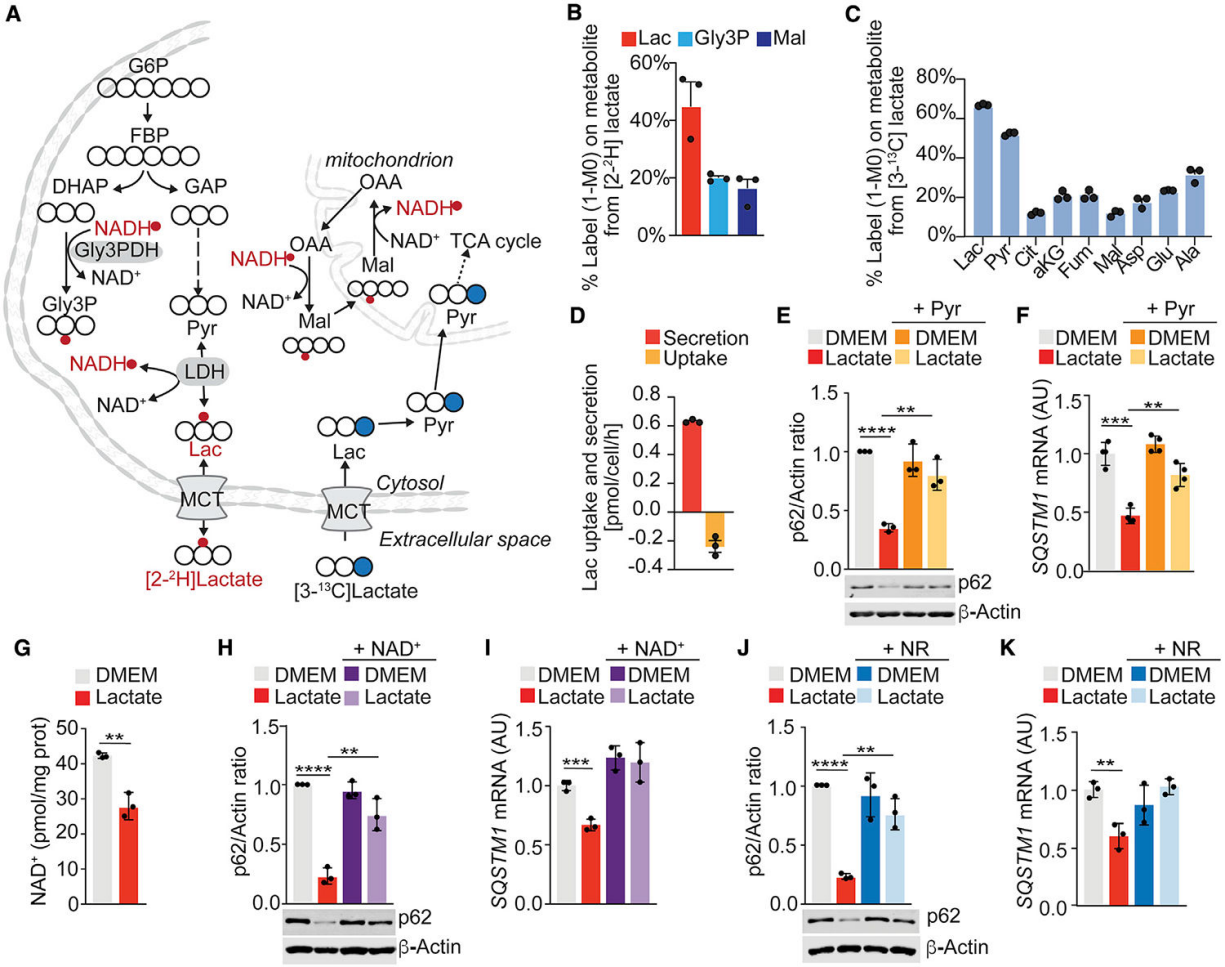
Changes in NAD^+^/NADH levels by lactate mediate p62 downregulation (A) Schematic depicting ^2^H (red) and ^13^C (blue) labeling from lactate tracer. Closed blue circles represent [^13^C]carbon; open circles represent [^12^C]carbon atoms; red circles represent ^2^H on metabolite. (B and C) Intracellular labeling on metabolites from [2-^2^H] (B) or [3-^13^C]lactate (C) in WPMY-1 cells cultured for 24 h in the presence of 10 mM labeled lactate (n = 3 biological replicates). (D) [^13^C]lactate uptake and [^12^C]lactate secretion in WPMY-1 cells cultured for 24 h in the presence of 10 mM [3-^13^C]lactate (n = 3 biological replicates). (E and F) Immunoblot analysis (E) and qPCR of *SQSTM1* (F) in WPMY-1 incubated with lactate or pyruvate for 48 h (n = 3 biological replicates). (G) Intracellular NAD^+^ levels in WPMY-1 cells treated or not with lactate (n = 3 biological replicates). (H and I) Immunoblot analysis (H) and *SQSTM1* mRNA levels (I) in WPMY-1 cells incubated with lactate and NAD^+^ (150 μM) for 48 h (n = 3 biological replicates). (J and K) Immunoblot analysis (J) and *SQSTM1* mRNA levels (K) in WPMY-1 cells incubated with lactate or NR (200 μM) for 48 h (n = 3 biological replicates). Results are shown as mean ± SEM. **p < 0.01, ***p < 0.001, ****p < 0.0001. MCT, monocarboxylate transporter. See also Figure S3.

We next interrogated the fate of the extracellular lactate in fibroblasts. We applied ^2^H and ^13^C isotopic tracing and mass spectrometry approaches to quantify the contribution of extracellular lactate to NADH and the central carbon metabolism. We cultured WPMY-1 cells in the presence of 10 mM [2-^2^H]lactate and determined the labeling of intracellular lactate, malate, and Gly3P. Extracellular lactate was metabolized by LDH activity, contributing hydrogen atoms to the NADH pool and regenerating the cytosolic nicotinamide adenine dinucleotide (NAD^+^) pool (Figures 4A and 4B). Next, we applied a [^13^C]lactate tracer and found approximately 20% label on tricarboxylic acid (TCA) cycle intermediates, indicating that extracellular lactate was metabolized as a carbon source fueling mitochondrial TCA cycle metabolism (Figures 4A and 4C). Experiments to determine lactate uptake and secretion fluxes in the presence of 10 mM exogenous [^13^C]lactate showed that WPMY-1 cells take up labeled lactate while secreting unlabeled lactate (Figures 4A and 4D). These results agree with the fact that lactate can be reversibly converted into pyruvate with the subsequent depletion of NAD^+^ levels in favor of the generation of NADH (Covarrubias et al., 2021). Increasing the levels of pyruvate in the culture media, which drives this reaction in the opposite direction, clearly impaired the ability of lactate to downregulate p62 (Figures 4E and 4F). Therefore, changes in the NAD^+^/NADH ratio could be instrumental for the mechanisms of action of lactate on the regulation of p62 levels. Consistent with this hypothesis, NAD^+^ levels in stromal fibroblasts treated with lactate were lower than in untreated cells (Figure 4G). Treatment with NAD^+^ or the NAD^+^ precursor, nicotinamide riboside (NR), completely rescued the lactate-induced reduction in p62, independently of autophagy (Figures 4H-4K, S3A, and S3B).

### PARP-1 inhibition by lactate downregulates p62 in stromal cells

**Figure 5.**
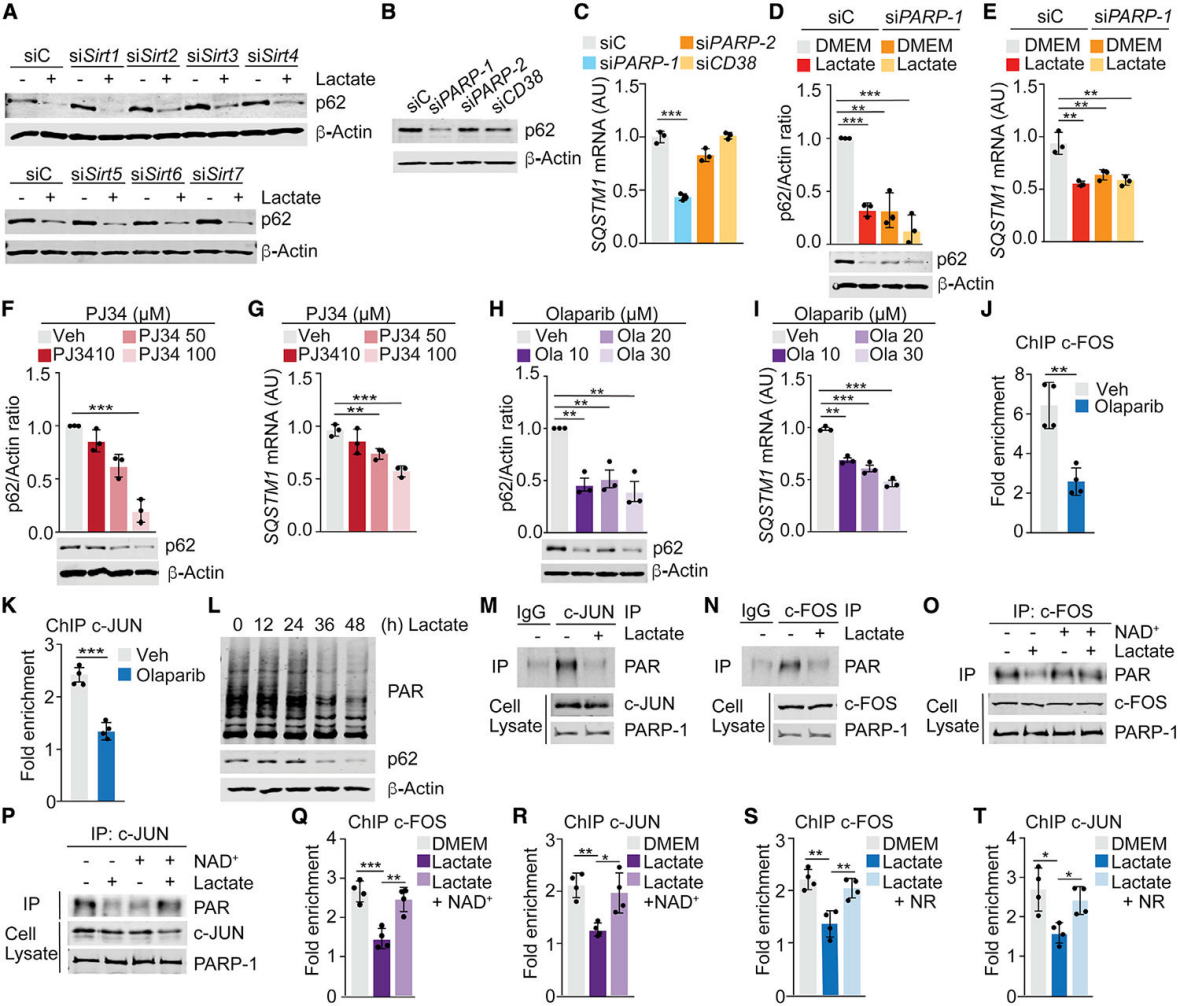
PARP-1 inhibition downregulates p62 levels in stromal cells (A) Immunoblot analysis in WPMY-1 cells transduced with the indicated siRNAs (n = 3 biological replicates). (B and C) Immunoblot analysis (B) and qPCR of *SQSTM1* (C) in WPMY-1 cells transduced with the indicated siRNAs (n = 3 biological replicates). (D and E) Immunoblot analysis (D) and qPCR of *SQSTM1* (E) in WPMY-1 cells transduced with the indicated siRNAs (n = 3 biological replicates). (F and G) Immunoblot analysis (F) and qPCR of *SQSTM1* (G) in WPMY-1 cells incubated with PJ34 for 24 h (n = 3 biological replicates). (H and I) Immunoblot analysis (H) and qPCR of *SQSTM1* (I) in WPMY-1 cells incubated with olaparib for 48 h (n = 3 biological replicates). (J and K) ChIP-PCR analysis of *SQSTM1* promoter (AP-1A) occupancy of c-FOS (J) or c-JUN (K) in WPMY-1 cells treated or not with olaparib (20 μM) for 48 h (n = 3 biological replicates). (L) Immunoblot analysis of PARylated protein levels in WPMY-1 cells incubated with lactate for the indicated times (n = 2 biological replicates). (M and N) PARylation of c-JUN (M) or c-FOS (N) in WPMY-1 treated or not with lactate for 48 h (n = 2 biological replicates). (O and P) PARylation of c-JUN (O) or c-FOS (P) in WPMY-1 treated or not with lactate and NAD^+^ for 48 h (n = 2 biological replicates). (Q and R) ChIP-PCR analysis of *SQSTM1* promoter (AP-1A) occupancy of c-FOS (Q) or c-JUN (R) in WPMY-1 cells treated or not with lactate and NAD^+^ for 48 h (n = 3 biological replicates). (S and T) ChIP-PCR analysis of *SQSTM1* promoter (AP-1A) occupancy of c-FOS (S) or c-JUN (T) in WPMY-1 cells treated or not with lactate and NR for 48 h (n = 3 biological replicates). Results are shown as mean ± SEM. *p < 0.05, **p < 0.01, ***p < 0.001. See also Figure S4.

NAD^+^ is a cofactor for three types of transcriptional and post-translational modulators, including sirtuins (SIRT1 through SIRT7), cyclic ADP-ribose synthases (CD38 and CD157), and PARPs (PARP-1/2) (Covarrubias et al., 2021). Of those, only the knockdown or CRISPR-Cas9 deletion of *PARP-1* fully mimicked the effect of lactate on p62 expression (Figures 5A-5E and S4A-S4E). Moreover, PARP-1 inhibitors PJ34 or olaparib also reduced the expression of p62 in a dose-dependent manner (Figures 5F-5I). Like lactate treatment, the effect of PARP-1 inhibition on p62 expression was detected in several stromal cell lines from different origins, with no effect on epithelial PCa cells (Figures S4F-S4J). PARP-1, in addition to its well-known role in DNA damage repair (DDR), also controls many other biological processes, including transcription (Feng et al., 2015). Thus, PARP-1 reportedly enhances the DNA binding and transactivation of many transcription factors (Gibson and Kraus, 2012). c-FOS and c-JUN have been previously shown to be poly(ADP-ribosyl)ated by PARP-1, which increased their DNA binding activity (Huang et al., 2009). Consistently, olaparib impaired the recruitment of c-FOS and c-JUN to the AP-1A binding site in the *SQSTM1* promoter (Figures 5J and 5K). These results suggest that the regulation of PARP-1 activity by changes in the levels of NAD^+^ triggered by lactate metabolism in stromal cells is instrumental for p62 downregulation. Therefore, we posited that lactate could affect the activation of PARP-1 and, consequently, the poly(ADP-ribosyl)ation of c-JUN and c-FOS. Consistently, lactate-treated cells have reduced total poly(ADP-ribosyl)ation levels and impaired poly(ADP-ribosyl)ation of c-JUN and c-FOS (Figures 5L-5N). Adding NAD^+^ to lactate-treated cells rescued the poly(ADP-ribosyl)ation of both transcription factors (Figures 5O and 5P) and their recruitment to the AP-1 regulatory site in the *SQSTM1* promoter (Figures 5Q and 5R). Similar results were obtained by treatment with NR (Figures 5S and 5T). These data demonstrate that depletion of NAD^+^ by lactate metabolism and the subsequent impairment of the poly(ADP-ribosyl)ation of c-FOS and c-JUN underlies the mechanism whereby lactate downregulates p62 in stromal fibroblasts.

### AP-1 is critical for CAF activation driven by p62 loss

**Figure 6.**
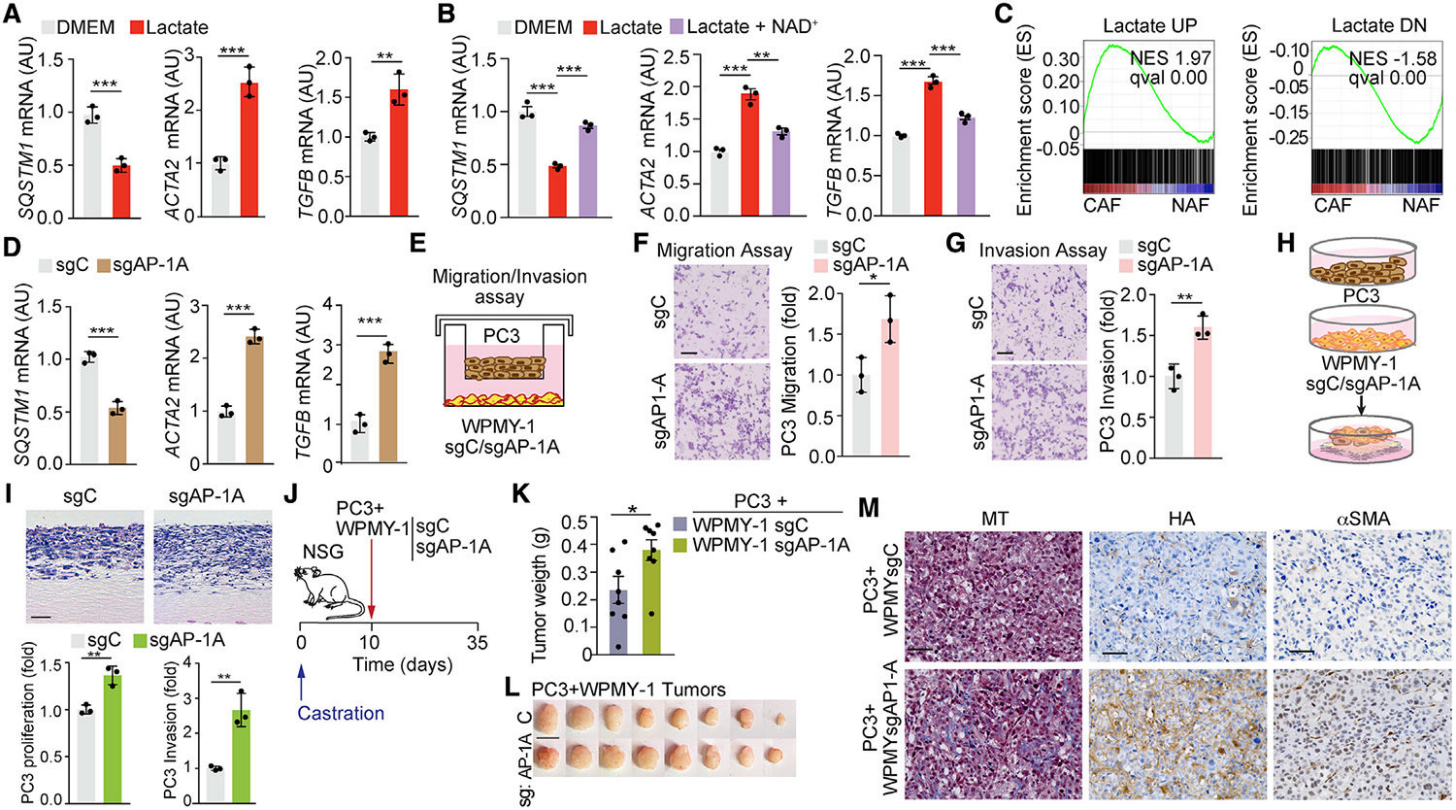
AP-1 control of the *SQSTM1* promoter in stromal cells is critical for PCa tumor growth and invasion (A and B) qPCR of mRNA levels of the indicated genes in WPMY-1 cells treated or not with lactate (A) and NAD^+^ (B) for 48 h (n = 3 biological replicates). (C) GSEA of “LACTATE UP” and “LACTATE DOWN” gene signatures in CAFs versus NAFs gene set GEO: GSE34312. (D) qPCR of mRNA levels of indicated genes in sgC and sgAP-1A WPMY-1 cells (n = 3 biological replicates). (E–G) Experimental design (E), migration (F), and invasion (G) of PC3 cells co-cultured with sgC or sgAP-1A WPMY-1 cells for 16 h. Representative images and quantification (n = 3 biological replicates). Scale bars: 50 μm. (H and I) Experimental design (H) and H&E staining (I) of organotypic gels combining PC3 cells with sgC and sgAP-1A WPMY-1 cells. Quantification of PC3 cells invasion and proliferation of experiments shown in (I) (n = 3 biological replicates). Scale bars: 100 μm. (J–M) Subcutaneous xenograft co-implantation in castrated NSG mice (male, 7 weeks old) of PC3 PCa cells with sgC or sgAP-1A WPMY-1 cells (PC3 + sgC WPMY-1: n = 8; PC3 + sgAP-1A WPMY-1: n = 8). Experimental design (J); tumor weight (K); gross images (L); and immunohistochemistry (IHC) for Masson’s trichrome, HA, and αSMA (M). Scale bars: 1 cm (L); 50 μm (M). Results are shown as mean ± SEM. *p < 0.05, **p < 0.01, ***p < 0.001. See also Figure S5.

We previously reported that the loss of p62 drives a CAF phenotype in stromal cells, which promotes tumor progression (Linares et al., 2017; Valencia et al., 2014). Because lactate secretion by PCa cells is necessary and sufficient to downregulate p62 in the stroma, we next determined whether lactate drives a CAF phenotype. Lactate treatment of fibroblasts increased the expression of bona fide markers of CAF activation, such as *ACTA2* and *TGF-β*, whose expression was reverted by NAD^+^ addition (Figures 6A and 6B). Gene set enrichment analysis (GSEA) of genes differentially expressed in lactate-treated cells showed enrichment in CAF signatures from a human stroma PCa dataset (GEO: GSE34312) (Figures 6C and S5A). Moreover, sgAP-1A fibroblasts in which the *SQSTM1* promoter was endogenously inactivated also showed increased expression of CAF markers (Figure 6D). Consistently, experiments in a two-chamber co-culture system demonstrated that sgAP-1A, but not sgC, fibroblasts promoted the migration and invasion of PC3 cells with no changes in proliferation (Figures 6E-6G and S5B). Similar results were obtained using a previously described 3D organotypic model that recapitulates key aspects of epithelial-stromal crosstalk in the TME (Valencia et al., 2014). Co-culture of PC3 cells with sgC and sgAP-1A fibroblasts in this organotypic assay demonstrated that sgAP-1A cells enhanced the invasiveness and proliferation index of PCa epithelial cells as compared with sgC cells (Figures 6H and 6I). Furthermore, subcutaneous xenograft co-implantation of PC3 cells with sgAP-1A fibroblasts resulted in increased tumor growth when compared with those co-implanted with sgC fibroblasts (Figures 6J-6L). Tumors generated with sgAP-1A fibroblasts showed increased markers of stromal activation, such as collagen deposition, hyaluronan (HA), and α-smooth muscle actin (α-SMA) (Figure 6M). These results establish that the AP-1-regulatory site in the *SQSTM1* promoter, which is the target of lactate-driven signals and has a pivotal role in the control of endogenous p62 levels in stromal cells, is critical for the induction of the CAF phenotype.

### PARP-1 inhibitors promote a desmoplastic response in stromal fibroblasts through p62

**Figure 7.**
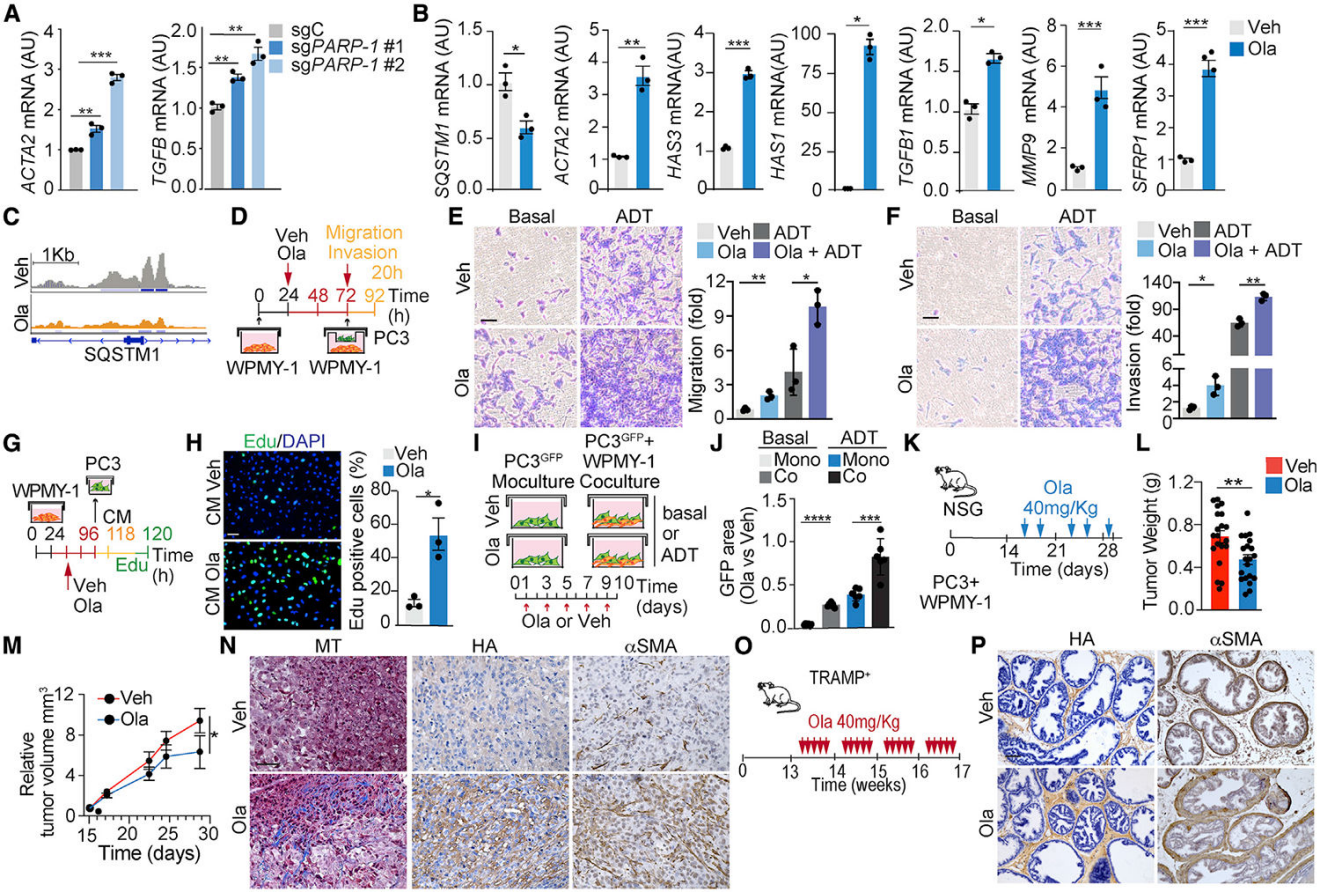
Olaparib treatment mimics lactate loss of p62 and promotes stromal activation (A and B) qPCR of mRNA levels of indicated genes in sgC and sg*PARP-1* WPMY-1 cells (A) or olaparib-treated cells (B) (n = 3 biological replicates). (C) Integrative Genomics Viewer (IGV) of ATAC-seq peaks in *SQSTM1* promoter in olaparib-treated WPMY-1 cells in basal conditions. (D–F) Transwell migration and invasion assays. Experimental design (D); transwell migration and invasion assay of PC3 PCa cultured with olaparib-pretreated WPMY-1 cells for 20 h (E and F, left). Scale bar: 50 μm. (E and F, right) Relative number of migrating and invading cells (n = 3 biological replicates). (G and H) Click-iT 5-ethynyl-2’-deoxyuridine (EdU) incorporation assay. Experimental design (G); representative images of EdU incorporation in PC3 PCa cells treated with CM from olaparib-treated WPMY-1 cells for 24 h (H, left); percentage of EdU-positive cells (n = 3 biological replicates) (H, right). Scale bar: 50 μm. (I and J) Survival assay of GFP-labeled PC3 PCa cells co-cultured with WPMY-1 cells or alone, treated with DMSO or olaparib, in basal and ADT conditions. Experimental design (I) and relative GFP-positive area after 10 days of culture (n = 6 biological replicates) (J). (K–N) Subcutaneous xenograft co-implantation in NSG mice (male, 7 weeks old) of PC3 PCa cells with WPMY-1 cells (PC3 + WPMY-1: n = 20). Mice were treated twice a week with olaparib 40 mg/kg for 2 weeks. Experimental design (K); tumor weight (g) (L); tumor volume (mm^3^) (M); and IHC for Masson’s trichrome, HA, and αSMA (N). Scale bars: 50 μm. (O and P) TRAMP^+^ mice treated with olaparib 40 mg/kg, 5 days a week for 4 weeks. Experimental design (O); IHC for HA and αSMA (P). Scale bars: 50 μm. Results are shown as mean ± SEM. *p < 0.05, **p < 0.01, ***p < 0.001. See also Figure S6.

According to our model, lactate triggers the downregulation of p62 in stromal fibroblasts through PARP-1 inhibition. Consistently, deletion of *PARP-1* by CRISPR-Cas9 mimicked the effect of lactate and induced CAF activation markers such as *ACTA2* and *TGF-β* (Figure 7A). Therefore, we hypothesized that PARP-1 inhibitors such as olaparib would be sufficient to induce stromal activation. In keeping with this, GSEA of RNA sequencing (RNA-seq) data from olaparib-treated fibroblasts, under basal or androgen deprivation (ADT) conditions, demonstrated enrichment in signatures of ECM remodeling, CAF activation, and well-established gene signatures for stromal activation in several types of cancer, including PCa (Figures S6A-S6C). Consistently, olaparib reduced *SQSTM1* mRNA and upregulated CAF markers such as *ACTA2*, *SFRP1*, *TGF-β*, *MMP9*, and HA synthases (Figure 7B). Furthermore, ATAC-seq analysis of olaparib-treated cells revealed a strong decrease in chromatin accessibility across the genome, in regions with consensus motifs for AP-1 transcription factors including the *SQSTM1* promoter, similarly to lactate treatment (Figures 7C, S6D, and S6E). Co-culture of PC3 cells with olaparib-pretreated WPMY-1 cells resulted in increased migration and invasion of PCa cells under basal conditions that was even more pronounced in ADT conditions (Figures 7D-7F). Moreover, olaparib-treated WPMY-1 CM led to increased PC3 proliferation (Figures 7G and 7H). Based on these observations, we posited that stromal activation in response to olaparib treatment could hamper its anti-tumor activity. To test this hypothesis, we co-cultured PC3 cells that were labeled with GFP (PC3^GFP^) either with (co-culture) or without (monoculture) WPMY-1 cells in basal or ADT conditions and treated or not with olaparib for 10 days (Figure 7I). Co-culture with WPMY-1 cells rendered PC3^GFP^ cells more resistant to olaparib as compared with treatment under monoculture conditions (Figure 7J). This effect was even more apparent under ADT conditions (Figure 7J). To investigate the pro-CAF phenotype of olaparib *in vivo*, we next co-implanted PC3 and WPMY-1 cells in NOD scid gamma (NSG) mice and treated them with either vehicle or olaparib (Figure 7K). *In vivo* olaparib treatment efficiently reduced tumor growth (Figures 7L-7M), consistent with previous reports (Li et al., 2017; Zhang et al., 2018), but also produced a strong desmoplastic response, characterized by enhanced collagen and HA deposition and increased αSMA (Figure 7N). A similar olaparib-induced desmoplastic response was found in the endogenous TRAMP mouse model (Figures 7O and 7P). To test the human relevance of these findings, we extended our analysis to two human PCa xenografts (VCAP and H660) and a PDX model previously reported to respond to olaparib treatment (Li et al., 2017; Zhang et al., 2018). Consistent with the mouse data, olaparib induced strong stromal activation in all human PCa models (Figure S6F).

## DISCUSSION

Despite recent advances in the characterization of the heterogeneity of fibroblast populations in the tumor stroma and the mounting evidence on their role in tumor progression, the molecular mechanisms that control stromal activation by the tumor epithelium remain largely unknown. Here, we have identified tumor-secreted lactate as the main signal from the epithelium that activates tumor fibroblasts by downregulating p62. Previous work from our laboratory and others has shown that p62 is a stromal tumor suppressor whose downregulation in fibroblasts is a key event for CAF activation (Duran et al., 2016; Goruppi and Dotto, 2013; Linares et al., 2017; Valencia et al., 2014). However, the mechanisms whereby p62 is downregulated by the tumor in the stroma were not understood. p62 regulation is subjected to a fine-tuned balance between transcription and post-translational mechanisms that control its degradation by autophagy or the proteosome machinery (Moscat and Diaz-Meco, 2009; Moscat et al., 2016). Here we show that lactate modulates p62 at the transcriptional level. Thus, although lactate reportedly induces autophagy (Brisson et al., 2016), which can contribute to lactate effects in tumor cells and the stroma, we show here that blocking autophagy or the proteosome failed to prevent p62 downregulation by lactate. At the transcriptional level, p62 has been shown to be regulated by several transcription factors, including AP-1, NRF2, NF-κB, and TFEB, under different conditions (Duran et al., 2008; Jain et al., 2010; Ling et al., 2012; Park et al., 2019). Here, we have identified the AP-1 binding site in the *SQSTM1* promoter as the critical element accounting for lactate-mediated p62 downregulation. Interestingly, these findings are reminiscent of our previously published data showing that AP-1 was also the major transcription factor for Ras-induced p62 mRNA expression in the tumor epithelium (Duran et al., 2008), which suggests that AP-1 is a hotspot for the upregulation of p62 by oncogenic transformation in the epithelium and its downregulation in the stroma. Regarding the composition of the AP-1 transcription factors mediating this effect in the stroma, our data identified c-FOS and c-JUN, but not FOSB or JUNB, in contrast with previous reports demonstrating that JUNB mediated repression of p62 in keratinocytes (Sukseree et al., 2021), which suggests that different AP-1 proteins may play distinct roles in p62 regulation under different cellular contexts.

Lactate has been considered for years as just the end product of glycolysis and, as such, a metabolic waste (Ippolito et al., 2019). However, more recent evidence has recognized many roles for lactate, including its relevance as a carbon source for cellular metabolism or as a signaling molecule in the TME (Brooks, 2018; Ippolito et al., 2019). Lactate is produced from pyruvate and is exported to the TME, where it can reach high concentrations of up to 40 mM, especially in tumor tissues (Walenta et al., 2000). Most tumor cells can engage in secretion and utilization of lactate depending on the context and extracellular microenvironment (Brooks, 2018). In addition to the shuttling that can take place among different tumor cells, lactate accumulation in the TME has significant effects on the non-malignant compartment, including, as shown here, on the activation of the fibroblasts surrounding the tumor, which results in their acquisition of the CAF phenotype (Bhagat et al., 2019). Therefore, targeting lactate metabolism as a potential therapeutic approach needs to integrate its effects not only on the tumor cell but also on the TME.

Lactate dehydrogenase catalyzes the conversion of lactate into pyruvate, which reduces NAD^+^, an important metabolic and signaling cofactor often dysregulated in cancer (Chini et al., 2021; Fang et al., 2017). Here we show that low NAD^+^ levels are central to the ability of lactate to downregulate p62 in fibroblasts and their CAF activation. Our data demonstrating that supplementation with NAD^+^ precursors, such as NR, rescued p62 levels and reverted the CAF phenotype established that altering the cell NAD^+^/NADH ratio could be a critical mechanism to modulate stromal activation. In keeping with this notion, downregulation of nicotinamide phosphoribosyl transferase (NAMPT), a rate-limiting enzyme in NAD^+^ synthesis, has been shown to promote renal and lung fibrosis with increased collagen deposition and ECM remodeling (Muraoka et al., 2019). Furthermore, recent evidence demonstrated that stromal nicotinamide *N*-methyltransferase (NNMT), an enzyme that impairs NAD^+^ biosynthesis by depleting its precursor *N*-nicotinamide, induces CAF differentiation and cancer progression (Eckert et al., 2019). This evidence suggests that the stromal activation promoted by NAD^+^ inhibition could limit the efficacy of anti-cancer therapies targeting NAD^+^ biosynthesis, such as NAMPT inhibitors, and could explain their reported limited efficacy *in vivo* (Galli et al., 2020). Supplementation with NR has been studied extensively, and its dietary administration is considered safe and effective to increase NAD^+^ levels (Martens et al., 2018). NR supplements are proposed to ameliorate inflammation and metabolic dysfunction in aging, and there is a renewed interest in the potential of NAD^+^-boosting therapies to treat human diseases (Chini et al., 2021; Fang et al., 2017). Based on our data, an additional benefit of increasing NAD^+^ levels might be to reprogram the stroma back to a less activated phenotype by restoring p62 levels. However, the role of NAD^+^ in cancer is complex, and manipulating its levels could be a double-edged sword (Chini et al., 2021; Fang et al., 2017). Thus, increasing the NAD^+^/NADH ratio by administering nicotinamide mononucleotide (NMN), another NAD^+^ precursor, has been shown to have a protumorigenic effect in K-Ras-driven pancreatic cancer (Nacarelli et al., 2019), unveiling the complexity of these therapeutic approaches.

Our work here identified PARP-1 inhibition as the key target of the reduced NAD^+^ levels in the transcriptional downregulation of p62 by lactate. PARP-1 is the major isoform of the PARP enzyme family and, like the other PARPs, its PARylation activity impacts multiple biological processes with a main dual function in DDR and transcriptional regulation (Feng et al., 2015; Gibson and Kraus, 2012). Most of the therapeutic strategies using PARP inhibitors rely on their blockade of DDR and the creation of synthetic lethality in combination with genetic defects in homologous recombination-mediated repair (Farmer et al., 2005; Lord and Ashworth, 2017; McCabe et al., 2006), which are the basis for the treatment of patients with BRCA1- and BRCA2-associated cancers (Mateo et al., 2019). The use of PARP inhibitors has been most successfully exploited in BRCA1/2-deficient breast and ovarian cancers (Sonnenblick et al., 2015), and recently olaparib has been approved for the treatment of metastatic castration resistant prostate cancer (mCRPC) in patients with DDR gene mutations (de Bono et al., 2020; Hussain et al., 2020). However, the fact that the percentage of mCRPC patients with these mutations who could benefit from that type of therapy is a minority has led to efforts for combinatorial strategies by chemically inducing DDR (Li et al., 2017; Zhang et al., 2018). Most of these efforts have been focused on the direct effect of PARP inhibitors on the tumor cell, and much less is known on how the tumor stroma responds to this type of treatment. Our data reported here showing that lactate inhibits PARP activity and that PARP-1 knockdown downregulates p62 through the impaired PARylation of AP-1 transcription factors, resulting in CAF activation, might have important therapeutic implications. Our results also highlight the increasingly recognized role of PARP-1 in transcriptional regulation as a key mechanism of action beyond its role in DDR (Feng et al., 2015; Liu et al., 2019) and identify an unanticipated function for these inhibitors in the activation of the pro-tumorigenic potential of the tumor stroma. These data are consistent with the previously reported protumorigenic phenotype of PARP-1 knockout mice in the context of the TRAMP^+^ model, indicating the dominant protumorigenic role of the TME in PCa progression under conditions of PARP-1 deficiency (Pu et al., 2014). This is a critical finding because CAF activation in the TME by PARP inhibitors might limit or even blunt their therapeutic efficacy. Therefore, a better understanding of the fundamental mechanisms controlling the activation of the stroma has the potential to identify vulnerabilities that can improve cancer therapies.

### Limitations of the study

Although our cell-based studies using CRISPR-Cas9 for PARP-1 and PARP-2 showed the selective role of PARP-1, but not PARP-2, in p62 regulation, the *in vivo* experiments used a PARP-1/2 inhibitor (olaparib) that does not discriminate between both PARP isoforms. *In vivo* selective strategies will be required to further characterize PARP isoform selectivity in p62 regulation and the acquisition of the CAF phenotype. Furthermore, our results demonstrate the contribution of c-FOS and c-JUN in p62 regulation, but further investigation will be necessary to elucidate the molecular identity of the AP-1 complex subjected to lactate control.

## STAR★METHODS

### RESOURCE AVAILABILITY

#### Lead contact

Further information and requests for resources and reagents should be directed to and will be fulfilled by the Lead contact, Maria T. Diaz-Meco (mtd4001@med.cornell.edu).

#### Materials availability

Cell and mouse lines generated in this study are available from the lead contact upon request with a completed Materials Transfer Agreement.

#### Data and code availability

- RNA-seq and ATAC-seq data have been deposited at GEO: GSE188720 and are publicly available as of the date of publication. Accession numbers are listed in the key resources table. Original raw data have been deposited at Mendeley and are publicly available as of the date of publication (https://doi.org/10.17632/xpzp3bv4ty.1). The DOI is listed in the key resources table. This paper analyzes existing, publicly available data. These accession numbers for the datasets are listed in the key resources table.
- This paper does not report original code.
- Any additional information required to reanalyze the data reported in this paper is available from the lead contact upon request.

### EXPERIMENTAL MODEL AND SUBJECT DETAILS

#### Mice

Animal handling and experimental procedures conformed to institutional guidelines and were approved by the Sanford-Burnham-Prebys Medical Discovery Institute Institutional Animal Care and Use Committee, and by the Weill Cornell Medicine Institutional Animal Care and Use Committee. For Olaparib treatment experiments, 13 week-old male TRAMP^+^ mice (C57BL/6-Tg(TRAMP) 8247 Ng/J, stock No: 003135) were purchased from The Jackson Laboratory, (Bar Harbor, ME, USA). TRAMP^+^ mice were generated in a C57BL/6 background and were born and maintained under pathogen-free conditions. All genotyping was done by PCR. Age-matched mice were used for all experiments. For xenograft experiments, 7-week-old male JAX NSG mice (572NCG) were purchased from Charles River Labs (Wilmington, MA, USA). NSG mice were purchased and maintained under pathogen-free conditions. All mice were maintained on food and water ad libitum and were age-matched and co-housed for all experiments. Mice were sacrificed and prostate, tumors or other organs were collected for analysis.

#### Cell lines

WPMY-1 (sex: male), PC3 (sex: male), DU145 (sex: male), TRAMPC2 (sex: male), PrEC (sex: male), RWPE1 (sex: male), LNCaP (sex: male), HEK293T (sex: female) and Phoenix-GP (sex: female) cell lines were purchased from ATCC. WPMY-1, PC3, DU145, TRAMPC2, HEK293T and Phoenix-GP were cultured in Dulbecco’s Modified Eagles Medium (DMEM, Corning). LNCaP, PrEC and RWPE1 cells were cultured in Roswell Park Memorial Institute Medium (RPMI, Corning). All base mediums were supplemented with 10% fetal bovine serum (FBS, Seradigm, Avantor), 2 mM glutamine (Corning) in an atmosphere of 95% air and 5% CO_2_. Androgen Deprivation Therapy conditions, cells were culture in RPMI media without phenol red (Gibco, Thermo Fisher) supplemented with 10% charcoal stripped FBS (Sigma), Glutamax (Thermo Fisher) and 100 U/mL penicillin 100 and 100 μg/mL streptomycin (Corning). Primary human fibroblasts: from lung, CAF WCM1630 (sex: female; age: 74 years old) and CAF WCM1674 (sex: female; age:); from breast, CAF WCM2793 (sex: female; age: 60 years old); from endometrium, NAF WCM2607A and CAF WCM2607A (sex: female; age: 64 years old), NAF WCM2573 and CAF WCM2573 (sex: female; age: 80 years old). Briefly, primary fibroblasts were isolated from either tumor or adjacent normal tissues. Tissue was first washed with PBS, cut into small pieces, and then digested with 0.004 g/mL collagenase IV for variable time depending on the tumor type. Afterwards, pieces of leftover digested tissue were transferred onto 6-well plates, fed with minimal DMEM supplemented with 10% FBS and 100 U/mL penicillin-streptomycin to obtain the fibroblasts. Fibroblast identity was confirmed by staining for αSMA, fibroblast activation protein (FAP), and S100A4 by flow cytometry. Cultures were tested weekly for mycoplasma contamination.

### METHOD DETAILS

#### Xenograft experiments

For mouse xenografts using PC3 PCa cells and sgC or sg*AP-1* WPMY-1 stromal cells. 7 weeks old NSG mice were surgically castrated and let androgen levels drop for 10 days. Cells were trypsinized washed two times in PBS and resuspend in DMEM. 1 × 10^6^ PC3 and 1 × 10^6^ sgC or sg*AP-1A* WPMY-1 for each mouse were resuspended in 100 μL DMEM:Matrigel (1:1) and injected subcutaneously into both flanks of immunocompromised NSG mice (PC3 + sgC WPMY-1 n = 4; PC3 + sgAP-1 WPMY-1 n = 4). Tumors were allowed to grow for 1 month. Mice were euthanized and tumors were collected and analyzed histologically. For Olaparib treatment in mouse xenografts. PC3 PCa cells and WPMY-1 stromal cells were trypsinized washed two times in PBS and resuspended in DMEM. 1 × 10^6^ PC3 and 1 × 10^6^ WPMY-1 for each mouse were resuspended in 100 μL DMEM:Matrigel (1:1) and injected subcutaneously into both flanks of immunocompromised NSG mice. Tumors were allowed to grow for 14 days, and mouse were randomly divided to receive vehicle, n = 10, or Olaparib (40 mg/kg/, 2 days each week, i.p.), n = 10. Tumors were measured twice a week. Mice were euthanized 14 days after the initiation of the treatment and tumors were collected and analyzed histologically. For Olaparib treatment in mice, 13 weeks-old male TRAMP^+^, n = 18, were randomly distributed to receive vehicle, n = 9, or Olaparib (40 mg/kg/, 5 days each week, i.p.) (Selleck Chemicals), n = 9. Mice were euthanized 1 month after the initiation of the treatment. Prostate and other organs were collected and analyzed histologically.

#### Cell culture experiments

To simulate the amounts of lactate that accumulates in the tumor microenvironment as consequence of the Warburg effect, WPMY-1 cells were incubated in culture medium with or without lactic acidosis (24 mM of Lactic acid buffered to pH 6.7–6.8 with NaOH) for 48h. To avoid fluctuations in extracellular pH during the Lactate treatment, WPMY-1 cells were grown in a bicarbonate-free DMEM (Gibco, Thermo Fisher) with 30 mM of HEPES (Gibco, Thermo Fisher) in absence of CO_2_. To knock out *PARP-1* in WPMY-1 cells, single-guide RNA sequences targeting *PARP-1* exon2 were purchased from Synthego and transduced into WPMY-1 cells with recombinant Streptococcus pyogenes Cas9 protein (Truecut Cas9 Protein v2, Thermo Fisher), using the Neon Transfection System 1 (Invitrogen) following the manufacturer’s protocol and single clones were expanded and screened by protein immunoblotting. To perform *AP-1A* binding site editing in the *SQSTM1* promoter in WPMY-1 cells, single-guide RNA sequences targeting the human AP-1A binding site (Synthego) was transduced into cells with a Cas9 protein and a single-stranded donor oligonucleotide (ssODN, IDT) using Neon Electroporation System. Single clones were expanded and screened for *AP-1A* editing by Sanger sequencing. A point mutation to disrupt the AP-1 enhancer element in the p62 promoter was introduced by Site Directed Mutagenesis (Stratagene). Knockdown of *MCT1, MCT4, FOS, PARP-1, PARP-2, CD38, ATG5, and SIRT1-7* genes in WPMY-1 cells were achieved by siRNA transfection using Lipofectamine RNAiMAX Transfection Reagent (Invitrogen). siRNAs used in this study are listed in Table S2. Transient overexpression was achieved by transfection using X-tremeGENE HP transfection reagent (Roche). Transfected cells were examined 48 h after transfection. Establishing stably GFP or RFP expressing cells was achieved by lentivirus-mediated transduction. Lentiviruses were produced in HEK293T cells using X-tremeGENE HP transfection reagent (Roche). Virus-containing supernatants were collected 48, 72 and 96 h after transfection, filtered to eliminate cells, and supplemented with 8 μg/mL polybrene. Cells were infected with three rounds of viral supernatants and selected with puromycin (3 μg/mL). For autophagy or proteasome inhibition, WPMY-1 cells were treated for 12 h with 100 nM bafilomycin A1 (Selleck Chemicals) or 12 h with 20 μM of MG132 (Sigma), respectively, or vehicle (DMSO). For PARP-1 inhibition, WPMY-1 cells were treated with PJ34 (2 days) or Olaparib (4 days) at the doses indicated in each experiment. For MCT1 inhibition, WPMY-1 cells were treated for 48h with 10 μM of AZD3965 (Selleck Chemicals). For c-JUN inhibition, WPMY-1 cells were treated for 48h with 10 μM of SP600125 (Selleck Chemicals) or 2 μM of JNK-IN-8 (Selleck Chemicals). For calpains and caspases inhibition, WPMY-1 cells were treated for 48h with 1–20 μM of Calpeptin (Tocris) or z-VAD-FMK (Tocris), respectively. For protein synthesis inhibition, WPMY-1 cells were treated with 50 μg/mL of cycloheximide (Sigma). Conditioned media (CM) was generated by collecting supernatant on day 3. CM was transferred to a 15 mL BD Falcon tube and centrifuged at 1300 rpm for 10 min. The supernatant was sterile filtered using a 22 mm filter (Millex-GV) with a 10 mL syringe barrel. The samples were stored at 20°C for future experiments. Fractionation of the CM was achieved using Amicon Ultra centrifugal filters (3KUltracel, Millipore). The supernatant was centrifuged at 4000 rpm for 1 h. The supernatant fraction that was >3 kDa remained above the filter and that which was <3 kDa passed through to the lower chamber. The >3 kDa fraction was resuspended in DMEM to the pre-filtration volume.

#### Organotypic cultures

Organotypic cultures were performed as described previously (Valencia et al., 2014). Briefly, gels were composed of one ml of a mixture of 1.75 volumes of Matrigel, 5.25 volumes of collagen type I (Corning), 1 volume of 1x DMEM, 1 volume of 10x DMEM, and 1 volume of filtered FBS. The mixture was plated onto 24-well plates coated with diluted collagen type I. Gels were allowed to equilibrate with 1 mL of 1x DMEM overnight at 37°C. 5 × 10^5^ cells PCa cells and prostate stromal cells (50:50) were then seeded on top of the matrix. Gel rafts were placed onto collagen-coated nylon sheets and lifted using a sterile supporting steel mesh to set up a raised air-liquid culture. Normal medium was changed in alternate days and organotypic cultures were allowed to grow for 14 days. Afterward, organotypic gels were harvested, fixed in 10% neutral buffered formalin, bisected, and embedded in paraffin. H&E stained sections were analyzed with a Zeiss light microscope supplemented with Axiovision40 software. Quantification of the invasion assays was performed as described previously (Valencia et al., 2014) using ImageProPlus software.

#### Migration and invasion assay

For co-culture migration and invasion assays, 8 × 10^4^ WPMY-1 cells were plated onto the lower chamber of a 24 well plate in DMEM containing 10% FBS (basal conditions) or 10% charcoal stripped FBS (ADT conditions) and vehicle (DMSO) or Olaparib 10 μM for 48 hours. 5 × 10^4^ PC3 were seeded in a transwell chamber (Corning Biocoat control inserts) or in a transwell invasion chamber (Corning BioCoat Matrigel Invasion Chambers), both with 8 μm membrane. PC3 were allowed to migrate or invade for 20 hours at 37°C, 5% CO_2_. Cells were fixed in cold methanol and stained with crystal violet.

#### Co-culture assays

Co-culture assays were performed as described previously (Valencia et al., 2014). Briefly, stromal cells were seeded in TC-treated 6-well plates and allowed to attach for 6 h before PCa cells were seeded on top of Milicell Cell Culture Inserts (Millipore). Inserts were then placed in the pre-seeded 6-well plate. For co-culture experiments of PC3-GFP label with WPMY-1 cells, 500 PC3 and 1000 WPMY-1 were seeded in a 96 well plate in DMEM containing 10% FBS (basal conditions) or 10% charcoal stripped FBS (ADT conditions) and vehicle (DMSO) or Olaparib 5 μM for 10 days. Media and treatments were refresh every 48 hours. Pictures were taken using EVOS M5000 Imaging System and GFP positive were measured using Fiji (Schindelin et al., 2012). For co-culture experiments of PC3-GFP label with WPMY-1 cells, 500 PC3 and 1000 WPMY-1 were seeded in a 96 well plate in DMEM containing 10% FBS (basal conditions) or 10% charcoal stripped FBS (ADT conditions) and vehicle (DMSO) or Olaparib 5 μM for 10 days. Media and treatments were refresh every 48 hours. Pictures were taken using EVOS M5000 Imaging System and GFP positive were measured using Fiji (Schindelin et al., 2012).

#### Isotopic labeling

WPMY-1 cells were cultured in DMEM medium supplemented with 10% FBS, 4 mM glutamine, 25 mM glucose, 30 mM HEPES, 10% FBS, 100 U/ml penicillin, and 100 μg/mL streptomycin in a humidified cell culture incubator at 37°C with no CO_2_. For isotopic tracing, cells were cultured for 48 h in growth medium, medium was then changed to growth medium containing 10 mM [3-^13^C]lactate (Cambridge Isotopes) or 10 mM [2-^2^H]lactate (Sigma). Cells were cultured in tracer media for 24 h. All media was adjusted to pH = 6.8.

#### Gas chromatography-mass spectrometry (GC/MS)

Metabolites were extracted using a modified Bligh and Dyer method and analyzed as previously described in detail (Linares et al., 2017). Briefly, intracellular metabolites were extracted with 0.25 mL–20°C methanol, 0.1 mL 4°C cold water, and 0.25 mL −20°C chloroform. The extracts were vortexed for 10 min at 4°C and centrifuged at 16,000×g for 5 min at 4°C. The upper aqueous phase was evaporated under vacuum at −4°C, the lower organic phase under airflow. To determine labeling on lactate in cell culture media, 10 μL of medium was extracted with 90 μL of extraction buffer consisting of 8 parts (v/v) methanol and 1 part (v/v) water, centrifuged at 16,000×g for 5 min at 4°C, and 60 μL was dried under vacuum. Derivatization for polar metabolites was performed using a Gerstel MPS with 15 μL of 2% (w/v) methoxyamine hydrochloride (Thermo Fisher) in pyridine (incubated for 60 min at 45°C) and 15 μL N-tertbutyldimethylsilyl-N-methyltrifluoroacetamide (MTBSTFA) with 1% tert-butyldimethylchlorosilane (Regis Technologies) (incubated further for 30 min at 45°C). Polar derivatives were analyzed by GC-MS using a DB-35MSUI column (30 m x 0.25 i.d. x 0.25 μm) installed in an Agilent 7890B gas chromatograph (GC) interfaced with an Agilent 5977B mass spectrometer (MS) operating under electron impact ionization at 70 eV. The MS source was held at 230°C, the quadrupole at 150°C, helium was used as carrier gas and the GC oven was held at 100°C for 1 min, increased to 300°C at 10°C/min, and held at 325°C for 3 min. Intracellular labeling on metabolites (corrected for natural abundance using in-house software) is depicted as 1-M0. YSI (yellow springs instrument) was used to quantify the concentration of media lactate and GC/MS to determine lactate labeling in cell culture media at 0 h and after 24 h of culture.

#### Measurement of NAD levels

Quantification was carried out using the NAD^+^/NADH quantification colorimetric kit according to the manufacturer’s protocol. At least three independent measurements were carried out.

#### Luciferase assay

Subconfluent cultures were transfected with Lipofectamine Plus (Invitrogen) with 100 ng of a *SQSTM1*-luciferase reporter gene plasmid and 2 ng of the *Renilla* control reporter pRL-CMV (Promega). After 48 h, cells were incubated with conditioned medium for 48 h. The level of promoter activity was evaluated by determining the firefly luciferase activity relative to renilla luciferase activity using the Dual Luciferase Assay System (Promega) according to the manufacturer’s instruction.

#### Histological analysis

Tissues from indicated mice were isolated, fixed in zinc buffered formalin overnight at 4°C, dehydrated, and embedded in paraffin. Sections (5 μm) were stained with hematoxylin and eosin (H&E). For immunohistochemistry, sections were deparaffinized, rehydrated, and then treated for antigen retrieval. After blocking in Protein Block Serum-Free solutions (DAKO), tissues were incubated with primary antibody overnight at 4°C followed by incubation with biotinylated secondary antibody. Endogenous peroxidase was quenched in 3% H_2_O_2_ in water for 10 min at room temperature. Antibodies were visualized with avidin-biotin complex (Vectastain Elite; Vector Laboratories) using diaminobenzidine as the chromogen. Stained sections were analyzed with a Zeiss light microscope supplemented with Zen 3.3 Bule edition software.

#### Immunoblotting analysis

Cells for protein analysis were lysed in RIPA buffer (20 mM Tris-HCl, 37 mM NaCl_2_, 2 mM EDTA, 1% Triton X-, 10% glycerol, 0.1% SDS, and 0.5% sodium deoxycholate) with phosphatase and protease inhibitors. For immunoprecipitations, cells were lysed in IP lysis buffer (100 mM NaCl, 25 mM Tris, 1% Triton X-, 10% glycerol, with phosphatase and protease inhibitors) and immunoprecipitated with 25 μL of 50% slurry of protein Recombinant protein G-Sepharose 4B (Thermo Fisher). Immunoprecipitates were washed three times with lysis buffer, once with high salt (500 mM NaCl), and once more with lysis buffer. Protein concentration in lysates was determined by using Protein Assay Kit (Bio-Rad). Cell extracts and immunoprecipitated proteins were denatured, subjected to SDS-PAGE, transferred to PVDF membranes (GE Healthcare). After blocking with 5% nonfat dry milk in Tris-buffered saline and 0.1% Tween (TBS-T), the membranes were incubated with the specific antibodies (as listed in key resources table) overnight at 4°C. After 2 h incubation with the appropriate horseradish peroxidase-conjugated antibodies, the immune complexes were detected by chemiluminescence (Thermo Fisher) or Near-infrared fluorescence (LI-COR).

#### Autophagy and LC3 flux analysis

For LC3 flux analysis WPMY-1 cells, treated or not with Lactate or NAD^+^ for 48 h, were treated with lysosomal inhibitors (PI) (20 mM NH_4_Cl and 200 μM leupeptin) for 0, 2 and 4 h, After the incubation, cells were lysed and subjected to immunoblotting analyses. Amount of LC3-II was normalized to that of actin and autophagosome formation as: LC3II_4h_ - LC3II_2h_.

#### RNA extraction and analysis

Total RNA from mouse tissues, cells and cultured organoids was extracted using the TRIZOL reagent (Invitrogen) and the RNeasy Mini Kit (QIAGEN), followed by DNase treatment. After quantification using a Nanodrop 1000 spectrophotometer (Thermo Fisher), RNA was either processed for RNA-seq or reverse-transcribed using random primers and MultiScribe Reverse Transcriptase (Applied Biosystems). Gene expression was analyzed by amplifying 20 ng of the complementary DNA using the CFX96 Real Time PCR Detection System with SYBR Green Master Mix (BioRad) and primers described in Table S1. The amplification parameters were set at 95°C for 30 s, 58°C for 30 s, and 72°C for 30 s (40 cycles total). Gene expression values for each sample were normalized to the 18s RNA.

#### Chromatin immunoprecipitation analysis

WPMY-1 cells were fixed by adding directly to the culture medium formaldehyde (HCHO; from a 37% HCHO–10% methanol stock, Calbiochem) to a final concentration of 1%. After, 20 min of incubation with 125 mM glycine, cells were washed with ice-cold phosphate-buffered saline (PBS) and lysed with in 50 mM Tris pH 8.0, 10 mM EDTA, 1% SDS and protease inhibitors and incubated 30 min at 4°C. Chromatin was sheared in a COVARIS S220 Focused-ultrasonicator to yield DNA fragment sizes of 200–1000 base pairs, and diluted 10 times in dilution buffer (20 mM Tris pH 8.0, 2 mM EDTA, 1% triton X-100, 150 mM NaCl and protease inhibitors). Immunoprecipitations were carried out overnight at 4°C using the following protein A-antibodies complexes: c-FOS, FOSB, c-JUN, JUNB. Immunocomplexes were washed three times with buffer TSEI (20 mM Tris pH 8.0, 2 mM EDTA, 1% triton X-100, 150 mM NaCl, 0.1% SDS and protease inhibitors), three washes with buffer TSEII (20 mM Tris pH 8.0, 2 mM EDTA, 1% triton X-100, 500 mM NaCl, 0.1% SDS and protease inhibitors), one wash with buffer TSEIII (10 mM Tris pH 8.0, 250 mM LiCl, 1 mM EDTA, 1% NP40, 1% Deoxycholate and protease inhibitors) and one wash with TE pH 8.0. Immunocomplexes were extracted in TE containing 1% SDS, and protein–DNA cross-links were reverted by heating at 65°C overnight. DNA was extracted by using a PCR purification kit (Qiagen). One-tenth of the immunoprecipitated DNA was used in each PCR, for which the promoter-specific primers were used.

#### RNA-seq preparation and sequencing

Total RNA was extracted using Quick-RNA MiniPrep kit (Zymo Research). Libraries were prepared from 200 ng of total RNA using the QuantSeq 3′ mRNA-Seq Library Prep Kit FWD for Illumina from Lexogen, and optional UMIs (Vienna, Austria). Barcoded libraries were pooled, and single end sequenced (1X75) on the Illumina NextSeq 500 using the High output V2.5 kit (Illumina Inc., San Diego CA).

#### ATAC-seq library preparation and sequencing

The cell pellet was resuspended in 50 μL lysis buffer and then spun down 500 × g for 10 min at 4°C. The nuclei pellet was resuspended into 50 μL transposition reaction mixture containing Tn5 transposase from Nextera DNA Library Prep Kit (Illumina) and incubated at 37°C for 30 min. Then the transposase-associated DNA was purified using MinElute PCR purification kit (QIAGEN). To amplify the library, the DNA was first amplified for 5 cycles using indexing primer from Nextera kit and NEBNext High-Fidelity 2X PCR master mix. To reduce the PCR amplification bias, 5 μL of amplified DNA after the first 5 cycles was used to do qPCR of 20 cycles to decide the number of cycles for the second round of PCR. Usually, the maximum cycle of the second round of PCR is 5 cycles. Then the total amplified DNA was size selected to fragments less than 800 bp using SPRI beads. Quantification of the ATAC-seq library was done with QuBit. The size of the pooled library was examined by TapeStation. Barcoded ATAC-seq libraries were pooled and paired end sequenced (sX75) on the Illumina NextSeq 500 using the High output V2.5 kit (Illumina Inc., San Diego, CA).

#### Bioinformatics analysis

For RNA-Seq, sequencing Fastq files were uploaded to BaseSpace and processed with RNA-Seq Alignment App (Illumina) to obtain raw reads counts for each gene. For 3′RNA-Seq, read data was processed with the BlueBee Genomics Platform (BlueBee, San Mateo, CA). GenePattern (https://genepattern.broadinstitute.org/gp/pages/index.jsf) was used to collapse gene matrix files (CollapseDataset module) or to assess the statistical significance of differential gene expression (ComparativeMarkerSelection module for microarray data and DESeq2 module for RNA-seq data). Gene Set Enrichment Analysis (GSEA) was performed using GSEA v4.1.0 software (http://www.broadinstitute.org/gsea/index.jsp) using the default parameters, with customized signatures, using the default parameters with customized signatures. Preranked GSEA analysis was performed using default parameters with MSigDB c2.all.v7.0.symbols (C2) and c5.all.v7.0.symbols (C5) collections. Briefly, differential express genes between Olaparib treated cells and vehicle were obtain using DESeq2, genes were sorted by log_2_FC≥ ±0.5 and padj≥0.05. Ranked genes were used as input for GSEA Preranked analysis. For ATACSeq, FASTQ files from ATAC-seq reads were aligned to UCSC mm10 with Bowtie2 (bowtie2 –very-sensitive -x mm10 −1 FILE_merged_R1.fastq −2 FILE_merged_R2.fastq -X 1000 -p 12 ∣ samtools view -u - ∣ samtools sort - > FILE.bam). Peak calling was performed with MACS2 with a threshold of q < 0.05. Peaks were annotated with ChipSeeker R package. For motif enrichment analysis in the differential peaks between vehicle and Olaparib-treated cells, p values were calculated using findMotifsGenome.pl with -size given,-len 6,8,10,12 and -mask program of HOMER v4.10.3 (Heinz et al., 2010). within 1–2 Kb from the TSS. For footprinting analysis, *rgt-hint differential* command was used to infer transcription factor activity and to plot the results (Li et al., 2019). Preranked GSEA analysis was performed using default parameters with MSigDB c2.all.v7.0.symbols (C2) and c5.all.v7.0.symbols (C5) collections. Briefly, differential express genes between Olaparib treated cells and vehicle were obtain using DESeq2, genes were sorted by log_2_FC≥ ±0.5 and padj≥0.05. Ranked genes were used as input for GSEA Preranked analysis. For ATACSeq, FASTQ files from ATAC-seq reads were aligned to UCSC mm10 with Bowtie2 (bowtie2 –very-sensitive -x mm10 −1 FILE_merged_R1.fastq −2 FILE_merged_R2.fastq -X 1000 -p 12 ∣ samtools view -u - ∣ samtools sort - > FILE.bam). Peak calling was performed with MACS2 with a threshold of q < 0.05. Peaks were annotated with ChIPSeeker R package. For motif enrichment analysis in the differential peaks between vehicle and Olaparib-treated cells, p values were calculated using findMotifsGenome.pl with -size given, -len 6,8,10,12 and -mask program of HOMER v4.10.3 (Heinz et al., 2010). within 1–2 Kb from the TSS.

### QUANTIFICATION AND STATISTICAL ANALYSIS

All the statistical tests were justified for every figure. All samples represent biological replicates. Data are presented as the mean ± SEM. Statistical analysis was performed using GraphPad Prism 8 or R software environment (http://www.r-project.org/). Significant differences between groups were determined using a Student’s t-test (two-tailed) when the data met the normal distribution tested by D’Agostino test. If the data did not meet this test, a Mann-Whitney *U*-test was used. Differences between more than 3 groups were determined using one-way ANOVA test (parametric) or Brown-Forsythe and Welch ANOVA tests (nonparametric) followed by Dunnett post hoc test. If the data did not meet this test, a Mann-Whitney test was used. Differences in Kaplan Meier plots were analyzed by Gehan-Breslow-Wilcoxon test. The chi-square test or Fisher’s exact test was used to determine the significance of differences between covariates. Logistic regression analysis was employed to estimate univariate and multivariate odds ratio and 95% confidence interval (CI). Values of p < 0.05 were considered as significantly different.

## Supplementary Material

1

## Figures and Tables

**Table:** KEY RESOURCES TABLE

REAGENT or RESOURCE	SOURCE	IDENTIFIER
Antibodies		
Mouse anti-β-actin	Sigma-Aldrich	Cat# A1978; RRID: AB_476692
Rabbit anti-SQSTM1/p62	Cell Signaling Technology	Cat# 23214; RRID: AB_11157045
Rabbit anti-LC3	Cell Signaling Technology	Cat# 4108; RRID: AB_2137703
Rabbit anti-JNK	Cell Signaling Technology	Cat# 9252; RRID: AB_22550373
Rabbit anti-phospho-c-Jun	Cell Signaling Technology	Cat#2361; RRID: AB_490908
Mouse anti-c-FOS	Santa Cruz Biotechnology	Cat# sc-8047X; RRID: AB_627253
Mouse anti-MCT1	Santa Cruz Biotechnology	Cat# sc-365501; RRID: AB_10841766
Mouse anti-MCT4	Santa Cruz Biotechnology	Cat# sc-376101; RRID: AB_10989419
Mouse anti-c-JUN	Santa Cruz Biotechnology	Cat# sc-166540X; RRID: AB_2280720
Rabbit anti-c-JUN	Cell Signaling Technology	Cat# 9165; RRID: AB_2130165
Rabbit anti-c-FOS	Santa Cruz Biotechnology	Cat# sc-52; RRID: AB_2106783
Mouse anti-PARP-1	Trevigen	Cat# 4335-MC-100; RRID: AB_2572318
Mouse anti-PAR Polymer	Cell Signaling Technology	Cat# 4947; RRID: AB_823547
Mouse anti-Smooth muscle actin	Dako	Cat# M0851; RRID: AB_2223500
Mouse anti-Collagen I	Abcam	Cat# ab88147; RRID: AB_2081873
Goat anti-Mouse IgG1, secondary, HRP	Thermo Fisher Scientific	Cat# PA1-74421; RRID: AB_10988195
Goat anti-Rabbit IgG, secondary, HRP	Thermo Fisher Scientific	Cat# 31461; RRID: AB_228347
Goat anti-Mouse IgG1, secondary, Alexa Fluor 488	Thermo Fisher Scientific	Cat# A21121; RRID: AB_2535764
Donkey anti-Rat IgG, secondary, Alexa Fluor 488	Thermo Fisher Scientific	Cat# A21208; RRID: AB_2535794
Donkey anti-Rabbit IgG, secondary, Alexa Fluor 568	Thermo Fisher Scientific	Cat# A10042; RRID: AB_2534017
Goat anti-Rabbit IgG, secondary, IRDye 800	LI-COR Biosciences	Cat# 926-32211; RRID: AB_621843
Goat anti-Mouse IgG1, secondary, IRDye 800	LI-COR Biosciences	Cat# 926-32350; RRID: AB_2782997
Goat anti-Mouse IgG, secondary, IRDye 800	LI-COR Biosciences	Cat# 926-32210; RRID: AB_621842
Bacterial and virus strains		
DH5α Competent Cells	Thermo Scientific	Cat# 18265017
One Shot Stbl3 Chemically Competent	Thermo Scientific	Cat# C737303
Chemicals, peptides, and recombinant proteins		
X-tremeGENE transfection reagent	Roche	Cat# 6366236001
MG132	Sigma-Aldrich	Cat# 474790
Bafilomycin A1	Selleck Chemicals	Cat# S1413
Cyclohexamide	Sigma-Aldrich	Cat# C7698
PJ34	Selleck Chemicals	Cat# S7300
Olaparib	Selleck Chemicals	Cat# S1060
AZD3965	Selleck Chemicals	Cat# S7339
SP600125	Selleck Chemicals	Cat# S1460
Calpeptin	Selleck Chemicals	Cat# S7396
JNK-IN-8	Selleck Chemicals	Cat# S4901
β-Nicotinamide adenine dinucleotide (NAD)	Sigma-Aldrich	Cat# 10127981001
Nicotinamide riboside (NR)	Biosynth	Cat# N2555
Sodium Pyruvate	Sigma-Aldrich	Cat# P2256
L-Lactic Acid	MP	Cat# 219022805
[3-^13^C]lactate	Cambridge Isotopes	Cat# CLM-1578
[2-^2^H]lactate	Sigma-Aldrich	Cat# 693987
Dimethyl Sulfoxide	Fisher BioReagents	Cat# BP2311
Chloroform	Sigma-Aldrich	Cat# 288306
Methanol	Sigma-Aldrich	Cat# 1424109
Methoxyamine hydrochloride	Thermo Scientific	Cat# TS-45950
Pyridine	Thermo Scientific	Cat# P368-500
N-tertbutyldimethylsilyl-N-methyltrifluoroacetamide	Regis Technologies	Cat# 77377-52-7
ECL Western Blotting Substrate	Thermo Scientific	Cat# 32106
HEPES	Gibco	Cat# 15630080
L-Glutamine	Corning	Cat# 25-005-CI
Pencillin Streptomycin Solution, 100x	Corning	Cat # 30-002-CI
Lipofectamine 2000 Transfection Reagent	Invitrogen	Cat# 11668019
Lipofectamine RNAiMAX Transfection Reagent	Invitrogen	Cat# 13778030
Opti-MEM Reduced Serum Medium	Gibco	Cat# 31985070
PBS (no calcium, no magnesium)	Gibco	Cat# 10010-023
Puromycin	Omega Scientific, inc.	Cat# PR-01
Quick-RNA Miniprep Kit	Zymo Research	Cat# R1054
RNA*later* Stabilization Solution	Invitrogen	Cat# AM7021
TRIzol	Thermo Fisher Scientific	Cat# 15596018
DMEM	Corning	Cat# 10-017CV
DMEM, powder, high glucose	Gibco	Cat# 12800017
GlutaMAX Supplement	Gibco	Cat# 35050061
Avantor® Seradigm Select Grade Fetal Bovine Serum (FBS)	Seradigm, VWR	Cat# 89510-182
Charcoal stripped FBS	Sigma	Cat# F6765
Corning® Tail Collagen I	Corning	Cat # 354236
Polybrene Infection Reagent	Sigma-Aldrich	Cat# TR-1003-G
TrueCut Cas9 Protein v2	Thermo Fisher Scientific	Cat# A36498
Recombinant protein G-Sepharose 4B	Thermo Fisher Scientific	Cat# 101242
Critical commercial assays		
Neon™ 10 μL Electroporation Kit	Thermo Fisher Scientific	Cat# MPK1096
NAD/NADH Quantitation Colorimetric Kit	Biovision	Cat# K337
Corning Biocoat Control Inserts	Corning	Cat# 354578
Corning BioCoat Matrigel Invasion Chambers	Corning	Cat# 354483
Trichrome Stain (Masson) Kit	Sigma-Aldrich	Cat# HT15
Deposited data		
RNA-seq	This study	GEO: GSE188720
ATAC-seq	This study	GEO: GSE188720
3’ RNA-seq	This study	GEO: GSE188720
Raw Data	This study; Mendeley Data	https://doi.org/10.17632/xpzp3bv4ty.1
Experimental models: Cell lines		
Human WPMY-1	ATCC	Cat# CRL-2854, RRID: CVCL_3814
Human WPMY-1 (*sgAP-1A*)	This study	N/A
Human PC3	ATCC	Cat# CRL-1435, RRID: CVCL_A4BV
Human DU145	ATCC	Cat# HTB-81, RRID: CVCL_0105
Human LNCAP	ATCC	Cat# CRL-3315, RRID: CVCL_4782
Human RWPE1	ATCC	Cat# CRL-11609, RRID: CVCL_3791
Human PrEC	ATCC	Cat# PCS-440-010,
Mouse TRAMPC2	ATCC	Cat# CRL-2731, CVCL_3615
Human HEK293T	ATCC	Cat# CRL-3216, RRID: CVCL_0063
Human Phoenix-GP	ATCC	Cat# CRL-3215
Human Prostate Fibroblasts	Science Cell Research laboratories	Cat# 4430
Primary lung fibroblast CAF WCM1630	Olivier Elemento, Ph.D., WCM	N/A
Primary lung fibroblast CAF WCM1674	Olivier Elemento, Ph.D., WCM	N/A
Primary breast fibroblast CAF WCM2793	Olivier Elemento, Ph.D., WCM	N/A
Primary endometrium fibroblast NAF WCM2607A	Olivier Elemento, Ph.D., WCM	N/A
Primary endometrium fibroblast CAF WCM2607A	Olivier Elemento, Ph.D., WCM	N/A
Primary endometrium fibroblast NAF WCM2573	Olivier Elemento, Ph.D., WCM	N/A
Primary endometrium fibroblast CAF WCM2573	Olivier Elemento, Ph.D., WCM	N/A
Experimental models: Organisms/strains		
C57BL/6-Tg(TRAMP)8247Ng/J	The Jackson Laboratory	Stock No: 003135
NSG mice	Charles Rivers Labs	Stock No: 572NCG
Oligonucleotides		
Real-time PCR primers	This manuscript	Table S1
siRNA oligonucleotides and guides	This manuscript	Table S2
Recombinant DNA		
pWZL Blast GFP	Addgene	Cat# 12269
Lentiviral packaging plasmid psPAX2	Addgene	Cat# 12260
Lentiviral packaging plasmid pMD2.G	Addgene	Cat# 12259
LentiCRISPR v2	Addgene	Cat# 52961
Software and algorithms		
Graphpad Prism 8	Graphpad	https://www.graphpad.com/scientificsoftware/
ImageJ	NIH	https://imagej.nih.gov/ij/
RStudio (1.1.456)	R Core Team	https://www.r-project.org/
R	R Core Team	https://www.r-project.org/
GSEA (v4.1.0)	Broad Institute	http://www.broadinstitute.org/gsea/index.jsp
BaseSpace	Illumina	https://basespace.illumina.com/
Morpheus	Broad Institute	https://software.broadinstitute.org/morpheus/
NextBio	Illumina	https://nextbio.com
GenePattern	Broad Institute	https://cloud.genepattern.org/gp/pages/login.jsf
Lexogen QuantSeq DE 1.3.0	BlueBee Cloud	https://www.bluebee.com
Bowtie2-2.3.4.3	(Langmead and Salzberg, 2012)	N/A
Other		
EVOS FL Auto Imaging System	Thermo Fisher Scientific	N/A
EVOS M5000 Imaging System	Thermo Fisher Scientific	N/A
NanoDrop 1000 spectrophotometer	Thermo Fisher Scientific	N/A
Zeiss LSM 710 NLO Confocal Microscope	Carl Zeiss Microscopy	N/A
Nikon A1R HD Confocal Microscope	Nikon	N/A
Neon™ Transfection System	Thermo Fisher Scientific	Cat# MPK5000

## Highlights

- Tumor-secreted lactate downregulates p62 in stromal fibroblasts
- Lactate reduces NAD^+^ levels and PARP-1 activity to downregulate stromal p62
- AP-1 is critical for lactate-induced p62 loss in stromal fibroblasts
- PARP-1 inhibitors promote a desmoplastic response in stromal fibroblasts through p62
