# Supplementary material for: The lactate-NAD+ axis activates cancer-associated fibroblasts by downregulating p62
Source: Cell Rep. Author manuscript; Available in PMC 2022 May 27. (PMC9136538; doi:10.1016/j.celrep.2022.110792)
Supplement: 1 [file NIHMS1806835-supplement-1.pdf]

Cell Reports, Volume 39

Supplemental information

The lactate-NAD$^+$ axis activates cancer-associated fibroblasts by downregulating p62

Juan F. Linares, Tania Cid-Diaz, Angeles Duran, Marta Osrodek, Anxo Martinez-Ordoñez, Miguel Reina-Campos, Hui-Hsuan Kuo, Olivier Elemento, M. Laura Martin, Thekla Cordes, Timothy C. Thompson, Christian M. Metallo, Jorge Moscat, and Maria T. Diaz-Meco

Supplemental Information

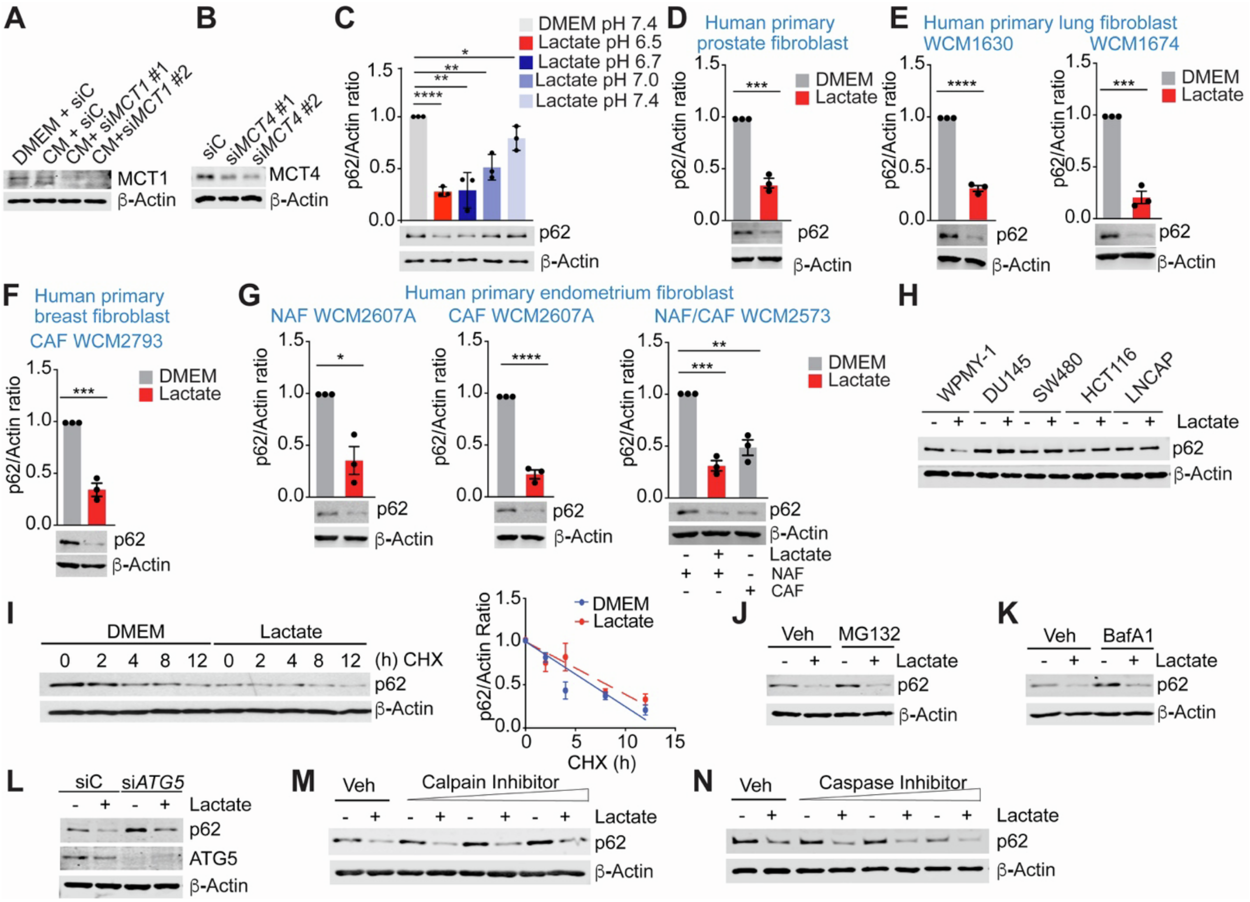

Figure S1. Lactate-driven p62 downregulation is not affected by different degradative mechanisms, Related to Figure 2

(A) Immunoblot analysis for the indicated proteins in WPMY-1 cells, transduced with the indicated siRNAs, incubated with CM from PC3 cells for 48 h. (B) Immunoblot analysis for the indicated proteins in PC3 cells, transduced with the indicated siRNAs (C) Immunoblot analysis for the indicated proteins in WPMY-1 cells incubated with lactate 24 mM at different pH for 48 h (n= 3 biological replicates). (D-G) Immunoblot analysis for indicated proteins in primary prostate fibroblasts (D), lung primary fibroblasts (E), breast primary fibroblasts (F), and endometrial primary fibroblasts(G), treated or not with lactate for 48 h (n= 3 biological replicates). (H) Immunoblot analysis for indicated proteins in WPMY-1 and PCa cells treated or not with lactate for 48 h (n= 2 biological replicates). (I) WPMY-1 cells, incubated or not with lactate for 48 h, were incubated with 50 µg/ml of cycloheximide (CHX) and protein stability

was determined by immunoblotting at indicated time points. p62 levels were normalized to actin (n= 3 biological replicates). (J and K) Immunoblot analysis for indicated proteins in WPMY-1 cells treated or not with lactate and MG132 (J) or Bafilomycin A1 (K) for 48 h (n= 3 biological replicates). (L) Immunoblot analysis for indicated proteins in WPMY-1 cells, treated or not with lactate for 48 h, and transduced with the indicated siRNAs (n= 3 biological replicates). (M and N) Immunoblot analysis for indicated proteins in WPMY-1 cells, treated or not with lactate for 48 h and a dose-response of the Calpain inhibitor (M) or Caspase inhibitor (N) (n= 3 biological replicates). Results are shown as mean ± SEM. *$p < 0.05$, **$p < 0.01$, ***$p < 0.001$, ****$p < 0.0001$.

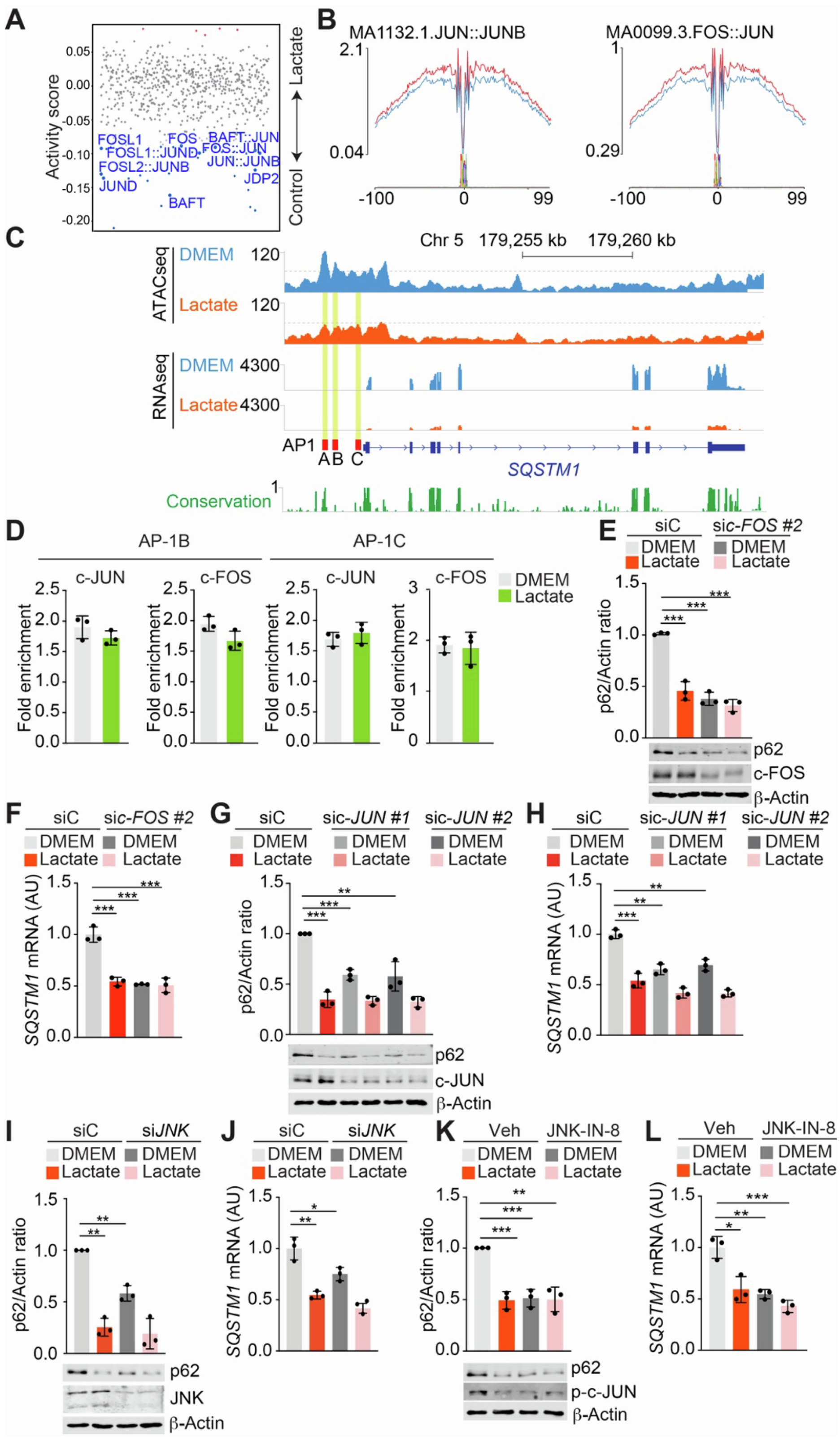

A
Activity score
Lactate
Control
FOSL1
FOSL1::JUND
FOSL2::JUNB
JUND
FOS
BAFT::JUN
FOS::JUN
JUN::JUNB
JDP2
BAFT
B
MA1132.1.JUN::JUNB
MA0099.3.FOS::JUN
C
Chr 5 179,255 kb 179,260 kb
ATACseq
RNAseq
DMEM
Lactate
AP1
A B C
SQSTM1
Conservation
D
AP-1B
AP-1C
c-JUN
c-FOS
Fold enrichment
DMEM
Lactate
E
siC
sic-FOS #2
p62/Actin ratio
p62
c-FOS
β-Actin
F
SQSTM1 mRNA (AU)
G
sic-JUN #1
sic-JUN #2
c-JUN
H
I
siJNK
JNK
J
K
Veh
JNK-IN-8
p-c-JUN
L

Figure S2. AP-1 controls p62 downregulation by lactate, Related to Figure 3

(A) Footprinting analysis of ATAC-seq data in lactate-treated WPMY-1 cells, top downregulated motifs are indicated. (B) Footprinting analysis of selective AP-1 family transcription factors in ATAC-seq data from (A). (C) Genome browser view at *SQSTM1* promoter. Chromatin accessibility (ATAC-seq) and RNA-seq profiles are shown in WPMY-1 cells treated or not with Lactate. (D) ChIP-PCR analysis of *SQSTM1* promoter (AP-1B or AP-1C) occupancy of c-JUN or c-FOS in WPMY-1 cells treated or not with lactate (24 mM) for 48 h (n= 3 biological replicates). (E and F) Immunoblot analysis for the indicated proteins (E) and qPCR of *SQSTM1* levels (F) in WPMY-1 cells incubated with lactate and transduced with the indicated siRNAs (n= 3 biological replicates). (G and H) Immunoblot analysis for the indicated proteins (G) and qPCR of *SQSTM1* levels (H) in WPMY-1 cells incubated with lactate and transduced with the indicated siRNAs (n= 3 biological replicates). (I and J) Immunoblot analysis for the indicated proteins (I) and qPCR of *SQSTM1* levels (J) in WPMY-1 cells incubated with lactate and transduced with the indicated siRNAs (n= 3 biological replicates). (K and L) Immunoblot analysis for the indicated proteins (K) and qPCR of *SQSTM1* levels (L) in WPMY-1 cells incubated with lactate and treated with 2 μM of JNK-IN-8 inhibitor (n= 3 biological replicates). Results are shown as mean ± SEM. *$p < 0.05$, **$p< 0.01$, ***$p < 0.001$.

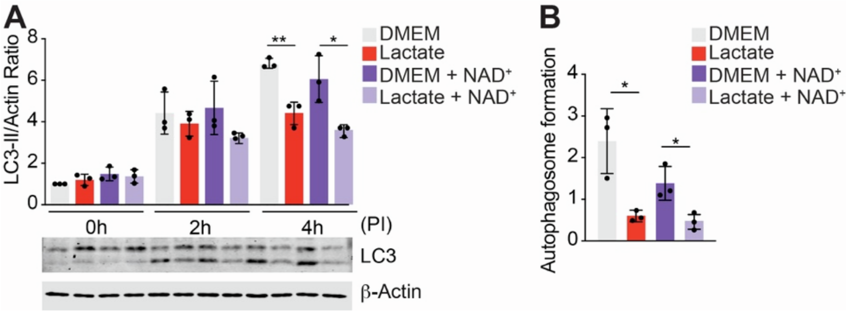

Figure S3. Autophagy is not involved in the lactate-mediated downregulation of p62, Related to Figure 4

(A) Immunoblot for LC3 in response to lysosomal inhibitors (PI) and quantification of LC3-II intensity normalized to actin in WPMY-1 cells, treated or not with lactate or NAD^+ for 48 h (n= 3 biological replicates). (B) Calculated speed of autophagosome formation from cells treated as in (A) (n= 3 biological replicates). Results are shown as mean ± SEM. *p < 0.05, **p< 0.01.

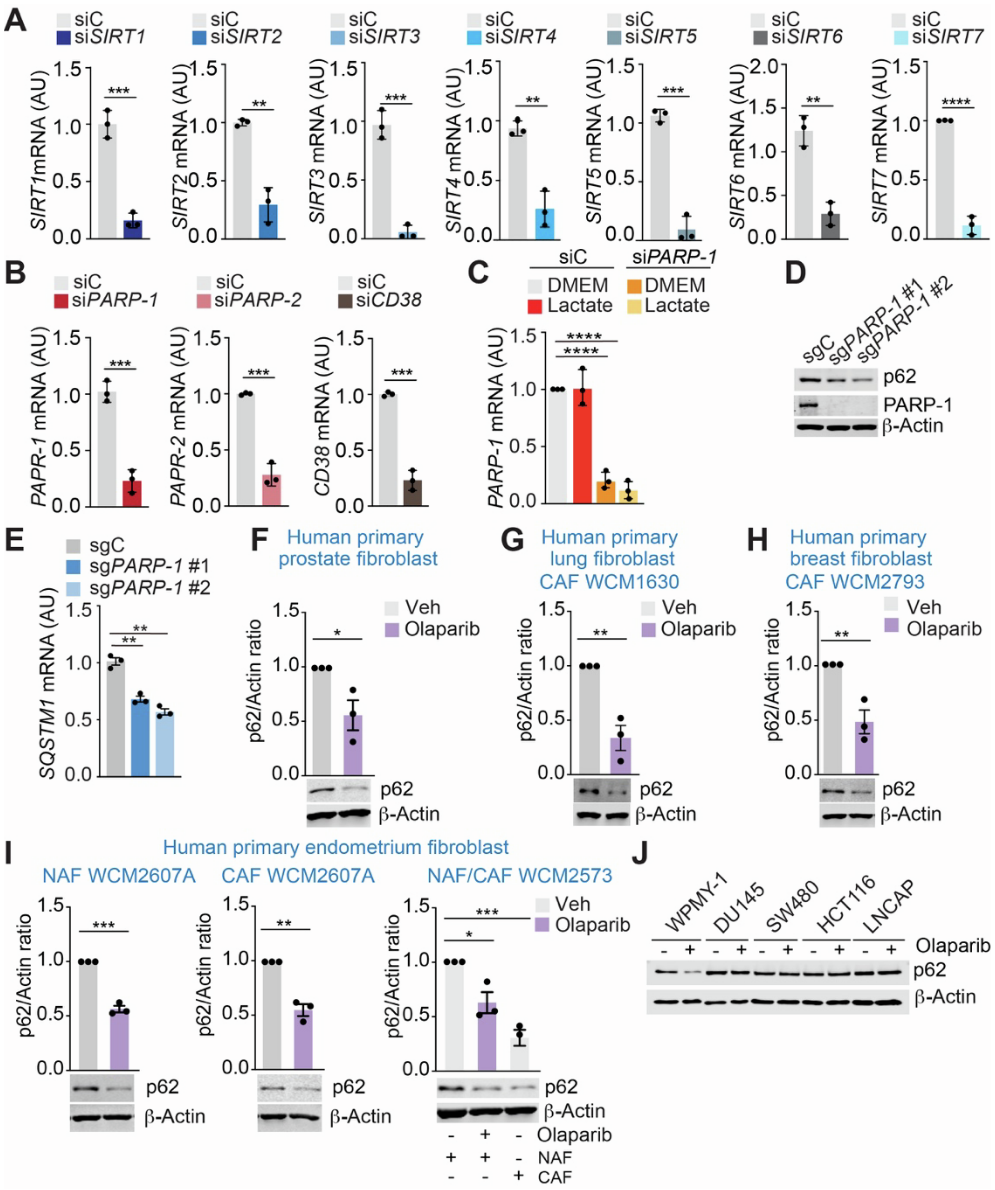

Figure S4. Selective downregulation of p62 by PARP-1 inhibition, Related to Figure 5

(A-C) qPCR analysis of mRNA levels of the indicated genes in WPMY-1 cells, transduced with the indicated siRNAs (n= 3 technical replicates). (D and E) immunoblot analysis for indicated proteins (D), and qPCR analysis of mRNA levels of the indicated genes (E) in sg*PARP-1* and sgC WPMY-1 cells (n= 3 biological replicates). (F-I) Immunoblot analysis for indicated proteins in primary prostate fibroblasts (F), lung primary fibroblasts (G), breast primary fibroblasts (H), and endometrial primary fibroblasts (I), treated or not with Olaparib, 20 µM, for 4 days (n= 3 biological replicates). (J) Immunoblot analysis for indicated proteins in WPMY-1 and PCa cells treated or not with Olaparib, 20 µM, for 4 days (n= 2 biological replicates). Results are shown as mean ± SEM. *$p < 0.05$, **$p < 0.01$, ***$p < 0.001$, ****$p < 0.0001$.

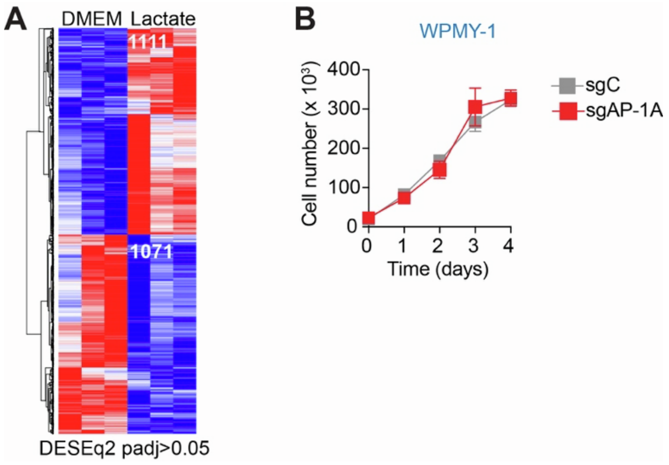

Figure S5. AP-1 is critical for CAF activation driven by p62 loss, Related to Figure 6

(A) Differential expressed genes heatmap of RNA-seq data of WPMY-1 cells treated or not with 24 mM lactate for 48 h. (B) Growth curves of sgC and sgAP-1A WPMY-1 cells (n= 3 biological replicates). Results are shown as mean ± SEM.

A

Olaparib vs Veh

Signature		NES	FDR
GO Collagen containing extracelluar matrix	Basal	3.00	0.00
	ADT	2.64	0.00
GO Extracellular matrix structural constituent	Basal	2.34	0.02
	ADT	2.15	0.07
GO External encapsulating structure	Basal	3.22	0.00
	ADT	2.75	0.03
GO Connective tissue development	Basal	1.90	0.10
	ADT	NA	NA
GO Cartilage development	Basal	1.57	0.24
	ADT	NA	NA
GO Collagen trimer	Basal	NA	NA
	ADT	1.80	0.19
GO Muscle contraction	Basal	NA	NA
	ADT	1.76	0.21
REACTOME Extracelullar matrix organization	Basal	2.02	0.01
	ADT	2.35	0.00
REACTOME Collagen byosinthesis and modifying enzymes	Basal	1.49	0.14
	ADT	1.57	0.12
REACTOME Collagen formation	Basal	1.37	0.22
	ADT	1.46	0.17
REACTOME Assembly of collagen fibrils and other multimeric structures	Basal	NA	NA

B

Olaparib vs Veh

Signature		NES	FDR
Herrera_CAF (GSE51257)	Basal	1.45	0.082
	ADT	1.42	0.130
Dakhova_Reactive Stroma (GSE9014)	Basal	1.54	0.009
	ADT	1.42	0.062
Tirosh_CAF (GSE103322)	Basal	1.11	NS
	ADT	1.18	0.210
Isella_CAF (GSE56710)	Basal	1.19	0.165
	ADT	1.56	0.002
Loda_Activated Stroma (GSE97284)	Basal	0.97	NS
	ADT	1.29	0.144
Asida_CAF (GSE34312)	Basal	1.35	0.000
	ADT	1.53	0.000
Lee_CAF (GSE132465,GSE132257)	Basal	1.59	0.000
	ADT	1.69	0.001

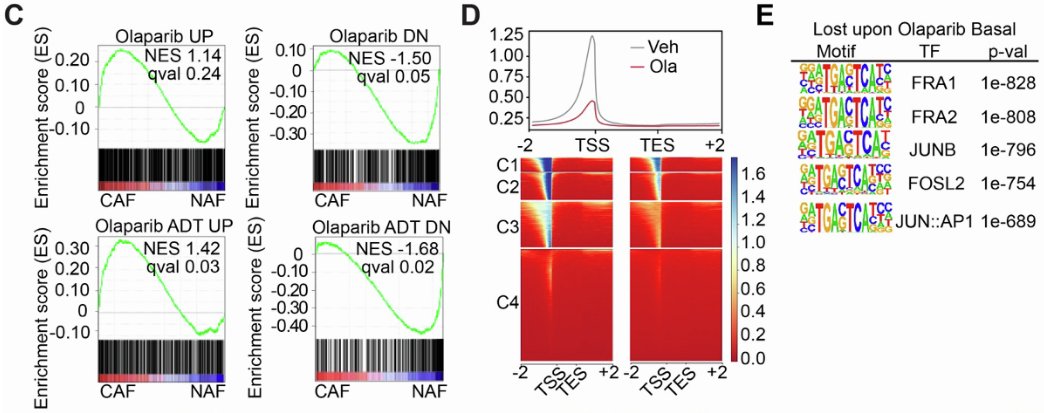

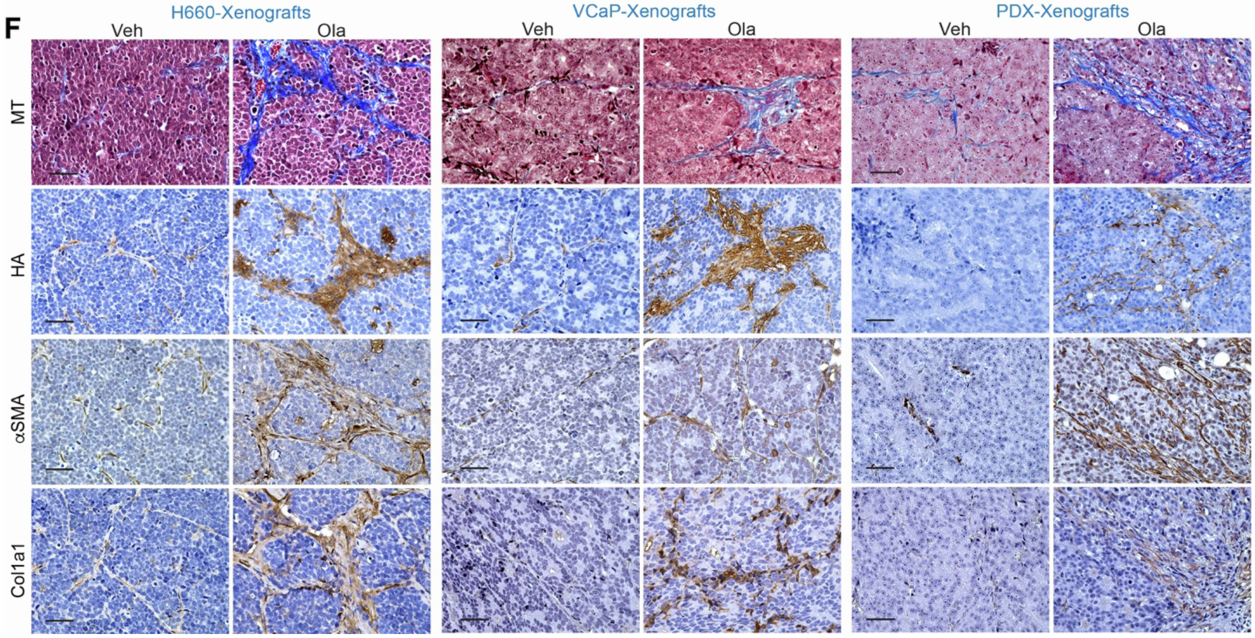

Figure S6. Olaparib promotes a desmoplastic response *in vitro* and *in vivo* in human prostate cancer xenograft, Related to Figure 7

(A) Enriched pathways obtained by GSEA pre-ranked analysis using DEG in Olaparib-treated WPMY-1 cells. (B) GSEA analysis for stromal activation and CAF phenotype gene signatures in Olaparib-treated WPMY-1 cells. (C) GSEA analysis of "Olaparib_UP", "Olaparib_DN", "Olaparib_ADT_UP", "Olaparib_ADT_DN" gene signatures in CAFs vs. NAFs gene set GSE34312. (D) Four clusters (C1, C2, C3 and C4) heatmap of ATAC-seq peaks, +/- 2 Kb from TSS, upon Olaparib treatment for 96 h in basal conditions. (E) HOMER discovery analysis of TFs enriched in promoter regions (1-2 Kb) closed upon Olaparib treatment in WPMY-1 cells (Veh vs Olaparib). (F) IHC for Masson Trichrome, HA, αSMA and Collagen1a1 (Col1a1) in Olaparib treated human PCa xenografts: human H660 xenografts, human VCaP xenografts, and human PCa PDX (MDA PCa 133-4), scale bars 50 μm.

Table S1. List of primers used, Related to STAR Methods

Gene symbol	Forward	Reverse
18s	5'- GTAACCCGTTGAACCCCAT-3'	5'- CCATCCAATCGGTAGTAGCG-3'
SQSTM1	5'- AGCGTCTGCGAGGGAAAG -3'	5'- ACCCGAAGTGTCCGTGTTT -3'
PARP-1	5'- TCTGCCTTGCTACCAATTCC-3'	5'- GATGGGTTCTCTGAGCTTCG -3'
PARP-2	5'- CAACACGGCTCCAGAAGACT -3'	5'- GCCTTCACAGATTCATCTTGCT -3'
CD38	5'- TGCTGATGACCTCACATGGT -3'	5'- CCATTGAGCATCACATGGAC -3'
MCT1	5'- TGTTCCTCTGTACTCTGGCC-3'	5'- GCAGTTTAGTAGCAAGCCCC-3'
MCT4	5'- CTCGCTCATCATGCTGAACC -3'	5'- ACACAGGAAGACAGGGCTAC-3'
ACTA2	5'- CGATAGAACACGGCATCATC -3'	5'- CATCAGGCAGTTCGTAGCTC-3'
TGFB1	5'- CGTGGAGCTGTACCAGAAATAC -3'	5'- CACAACTCCGGTGACATCAA -3'
HAS1	5'- GACTCCTGGGTCAGCTTCCTAAG-3'	5'- AAACTGCTGCAAGAGGTTATTCCT-3'
HAS3	5'- AGCACCTTCTCGTGCATCATGC -3'	5'- TCCTCCAGGACTCGAAGCATCT -3'
SFRP1	5'- TCAGGGGCTTCTTCTTCTTTG -3'	5'- TCTGAGGCCATCATTGAACA-3'
MMP9	5'- TCTTCCCTGGAGACCTGAGAAC -3'	5'- GACACCAAACTGGATGACGATG-3'
SIRT1	5'- TGCTGGCCTAATAGAGTGGCA-3'	5'- CTCAGCGCCATGGAAAATGT -3'
SIRT2	5'- CCATCTGTCACTACTTCATGC-3'	5'- AAGTCCTCCTGTTCCAGC-3'
SIRT3	5'- GCTGGACAGAAGAGATGC-3'	5'- GTGGATGTCTCCTATGTTACC -3'
SIRT4	5'- GCGTGTAAAAGAAGCCGACT-3'	5'- TTCTTCTCCCAGGCAGTCAG -3'
SIRT5	5'- CCCAGAACATCGATGAGC-3'	5'- GCCACAACTCCACAAGAGG-3'
SIRT6	5'- AGGGACAAACTGGCAGAGC -3'	5'- TTAGCCACGGTGCAGAGC-3'
SIRT7	5'- GCAGAGCAGACACCATCC-3'	5'- GTTCACGATGTAAAGCTTCG-3'
Chip-*AP-1A*	5'- ATCCCCCTATTACGACAGCG -3'	5'- GGCCTCCCGGAGGTAAACA -3'

Chip-*AP-1B*	5'- TCCCTCACCCTGCTCAGAC-3'	5'- CTGTGGCCTACAGACAGGTG-3'
Chip-*AP-1C*	5'-CCTCTCCCTGCACTGGGTA -3'	5'-ACCTGGGATCAGGGTACTGG-3'
Mutagenesis *AP-1A*	5'CGGTCATGGGACGCTCTGTCACTGCC GGCCAGACCACCTGACC-3'	5'GGTCAGGTGGTCTGGCCGGCAGTGAC AGAGCGTCCCATGACCG-3'
Mutagenesis *AP-1B*	5'CAGGCGCCTGGGCTGCTCTGTCACGC TTGGCCAGCACCTGTC-3'	5'GACAGGTGCTGGCCAAGCGTGACAGA GCAGCCCAGGCGCCTG-3'
Mutagenesis *AP-1C*	5'CCCAACTGAGGATATTGCTCTGTCAT GGCCAGGCCCAAGCC-3'	5'GGCTTGGGCCTGGCCATGACAGAGCA ATATCCTCAGTTGGG-3'

Table S2. List of siRNA oligonucleotides and guides, Related to STAR Methods

Oligonucleotides	Source	Identifier
Human *PARP-1* siRNA	Thermo Fisher	Cat# 10038
Human *MCT1* siRNA #1	Thermo Fisher	Cat# 106943
Human *MCT1* siRNA #2	Thermo Fisher	Cat# 106944
Human *MCT4* siRNA #1	Thermo Fisher	Cat# 107503
Human *MCT4* siRNA #2	Thermo Fisher	Cat# 107504
Human *c-FOS* siRNA #1	Thermo Fisher	Cat# 115631
Human *c-FOS* siRNA #2	Thermo Fisher	Cat# 115632
Human *c-JUN* siRNA #1	Thermo Fisher	Cat# 106741
Human *c-JUN* siRNA #2	Thermo Fisher	Cat# 115273
Human *JNK* siRNA	Thermo Fisher	Cat# 1320
Human *CD38* siRNA	Thermo Fisher	Cat# 119605
Human *PARP-2* siRNA	Thermo Fisher	Cat# 111561
Human *SIRT1* siRNA	Thermo Fisher	Cat# 136457
Human *SIRT2* siRNA	Thermo Fisher	Cat# 136455
Human *SIRT3* siRNA	Thermo Fisher	Cat# 136460
Human *SIRT4* siRNA	Thermo Fisher	Cat# 136462
Human *SIRT5* siRNA	Thermo Fisher	Cat# 19661
Human *SIRT6* siRNA	Thermo Fisher	Cat# 116148
Human *SIRT7* siRNA	Thermo Fisher	Cat# 116146
gRNA targeting human *PARP-1*<br>5'- GGGACTTTTCCATCAAACAT -3'	Synthego	N/A
gRNA targeting *AP1-A* in human *SQSTM1* promoter<br>5'- ATGGGACGCTGACTCACTGC-3'	Synthego	N/A
ssODN for human *SQSTM1* promoter editing<br>5'TCCCCAGCCCAGCCTCCAGGTAAGAGGTCACTGAGATGGGTGG CAGCAGGGGCCGGGGATCCCCCTATTACGACAGCGGTCATGGGA CGCCTGCCGGCCAGACCACCTGACCTCCGCGGCGGGAGGAGAGG GCC -3'	IDT	N/A
